# Supplementary material for: Non-linear association of liver enzymes with cognitive performance in the elderly: A cross-sectional study
Source: PLoS One. 2024 Jul 23;19(7):e0306839. doi: 10.1371/journal.pone.0306839 (PMC11265699; doi:10.1371/journal.pone.0306839)
Supplement: S1 Table — (DOCX) [file pone.0306839.s001.docx]

**Table S1** The cutoff points of the CERAD test, AFT, and DSST adjusted based on age.

| Age (years) | CERAD test score | AFT score | DSST score |
| --- | --- | --- | --- |
| ≥60 | 22 | 13 | 37 |
| ≥70 | 19 | 12 | 29 |

CERAD test: Consortium to Establish a Registry for Alzheimer's Disease test; AFT: animal fluency test; DSST: digit symbol substitution test.
