# Supplementary material for: Non-linear association of liver enzymes with cognitive performance in the elderly: A cross-sectional study
Source: PLoS One. 2024 Jul 23;19(7):e0306839. doi: 10.1371/journal.pone.0306839 (PMC11265699; doi:10.1371/journal.pone.0306839)
Supplement: S2 Table — (DOCX) [file pone.0306839.s002.docx]

**Table S2** Characteristics of liver enzymes and the CERAD test, AFT, and DSST among participants from NHANES 2011-2014 (N = 2764).

| Variablities | CREAD test | | | AFT | | | | DSST | | |
| --- | --- | --- | --- | --- | --- | --- | --- | --- | --- | --- |
|  | Normal cognitive performance | Low cognitive performance | *P* value | Normal cognitive performance | Low cognitive performance | | *P* value | Normal cognitive performance | Low cognitive performance | *P* value |
| Gender |  |  | <0.001 |  |  | | 0.830 |  |  | 0.099 |
| Male | 918 (45.2%) | 439 (60.1%) |  | 1000 (49.4%) | 357 (48.3%) | |  | 965 (46.7%) | 392 (56.1%) |  |
| Female | 1115 (54.8%) | 292 (39.9%) |  | 1025 (50.6%) | 382 (51.7%) | |  | 1100 (53.3%) | 307 (43.9%) |  |
| Age(years) | 68.7±6.5 | 71.1±7.0 | <0.001 | 68.7±6.5 | 71.3±6.8 | | <0.001 | 68.9±6.5 | 71.0±7.1 | <0.001 |
| ≥60 | 1119 (55.0%) | 379 (51.8%) |  | 1132 (55.9%) | 366 (49.5%) | |  | 1105 (53.5%) | 393 (56.2%) |  |
| ≥70 | 914 (45.0%) | 352 (48.2%) |  | 893 (44.1%) | 373 (50.5%) | |  | 960 (46.5%) | 306 (43.8%) |  |
| RACE |  |  | <0.001 |  |  | | <0.001 |  |  | <0.001 |
| Mexican American | 162 (8.0%) | 82 (11.2%) |  | 181 (8.9%) | 63 (8.5%) | |  | 141 (6.8%) | 103 (14.7%) |  |
| Other Hispanic | 175 (8.6%) | 102 (14.0%) |  | 187 (9.2%) | | 90 (12.2%) |  | 139 (6.7%) | 138 (19.7%) |  |
| Non-Hispanic White | 1049 (51.6%) | 302 (41.3%) |  | 1101 (54.4%) | 250 (33.8%) | |  | 1179 (57.1%) | 172 (24.6%) |  |
| Non-Hispanic Black | 441 (21.7%) | 186 (25.4%) |  | 385 (19.0%) | 242 (32.7%) | |  | 385 (18.6%) | 242 (34.6%) |  |
| Other Race | 206 (10.1%) | 59 (8.1%) |  | 171 (8.4%) | 94 (12.7%) | |  | 221 (10.7%) | 44 (6.3%) |  |
| Education status |  |  | <0.001 |  |  | | <0.001 |  |  | <0.001 |
| Below high school | 386 (19.0%) | 303 (41.5%) |  | 401 (19.8%) | 288 (39.0%) | |  | 291 (14.1%) | 398 (56.9%) |  |
| high school | 478 (23.5%) | 179 (24.5%) |  | 457 (22.6%) | 200 (27.1%) | |  | 498 (24.1%) | 159 (22.7%) |  |
| Above high school | 1169 (57.5%) | 247 (33.8%) |  | 1167 (57.6%) | 249 (33.7%) | |  | 1276 (61.8%) | 140 (20.0%) |  |
| Not recorded | 0 (0.0%) | 2 (0.3%) |  | 0 (0.0%) | 2 (0.3%) | |  | 0 (0.0%) | 2 (0.3%) |  |
| Poverty income ratio |  |  | <0.001 |  |  | | <0.001 |  |  | <0.001 |
| <1.3 | 474 (23.3%) | 273 (37.3%) |  | 473 (23.4%) | 274 (37.1%) | |  | 430 (20.8%) | 317 (45.4%) |  |
| ≥1.3 | 1397 (68.7%) | 391 (53.5%) |  | 1396 (68.9%) | 392 (53.0%) | |  | 1478 (71.6%) | 310 (44.3%) |  |
| Not recorded | 162 (8.0%) | 67 (9.2%) |  | 156 (7.7%) | 73 (9.9%) | |  | 157 (7.6%) | 72 (10.3%) |  |
| Body mass index(kg/m^2^) |  |  | 0.112 |  |  | | <0.001 |  |  | 0.009 |
| <25 | 528 (26.0%) | 206 (28.2%) |  | 529 (26.1%) | 205 (27.7%) | |  | 547 (26.5%) | 187 (26.8%) |  |
| 25-30 | 702 (34.5%) | 263 (36.0%) |  | 723 (35.7%) | 242 (32.7%) | |  | 724 (35.1%) | 241 (34.5%) |  |
| ≥30 | 779 (38.3%) | 246 (33.7%) |  | 756 (37.3%) | 269 (36.4%) | |  | 776 (37.6%) | 249 (35.6%) |  |
| Not recorded | 24 (1.2%) | 16 (2.2%) |  | 17 (0.8%) | 23 (3.1%) | |  | 18 (0.9%) | 22 (3.1%) |  |
| Physical activity level |  |  | <0.001 |  |  | | <0.001 |  |  | <0.001 |
| No | 798 (39.3%) | 371 (50.8%) |  | 756 (37.3%) | 413 (55.9%) | |  | 777 (37.6%) | 392 (56.1%) |  |
| Moderate | 355 (17.5%) | 117 (16.0%) |  | 366 (18.1%) | 106 (14.3%) | |  | 371 (18.0%) | 101 (14.4%) |  |
| Vigorous | 878 (43.2%) | 241 (33.0%) |  | 902 (44.5%) | 217 (29.4%) | |  | 914 (44.3%) | 205 (29.3%) |  |
| Not recorded | 2 (0.1%) | 2 (0.3%) |  | 1 (0.0%) | 3 (0.4%) | |  | 3 (0.1%) | 1 (0.1%) |  |
| Smoking status |  |  | 0.541 |  |  | | 0.260 |  |  | 0.001 |
| Non-smoker | 1010 (49.7%) | 352 (48.2%) |  | 997 (49.2%) | 365 (49.4%) | |  | 1031 (49.9%) | 331 (47.4%) |  |
| Former smoker | 776 (38.2%) | 267 (36.5%) |  | 774 (38.2%) | 269 (36.4%) | |  | 803 (38.9%) | 240 (34.3%) |  |
| Current smoker | 245 (12.1%) | 112 (15.3%) |  | 252 (12.4%) | 105 (14.2%) | |  | 229 (11.1%) | 128 (18.3%) |  |
| Not recorded | 2 (0.1%) | 0 (0.0%) |  | 2 (0.1%) | 0 (0.0%) | |  | 2 (0.1%) | 0 (0.0%) |  |
| Drinking status |  |  | 0.001 |  |  | | 0.004 |  |  | <0.001 |
| No | 474 (23.3%) | 190 (26.0%) |  | 455 (22.5%) | 209 (28.3%) | |  | 442 (21.4%) | 222 (31.8%) |  |
| Moderate | 973 (47.9%) | 312 (42.7%) |  | 969 (47.9%) | 316 (42.8%) | |  | 1059 (51.3%) | 226 (32.3%) |  |
| Heavy | 559 (27.5%) | 209 (28.6%) |  | 571 (28.2%) | 197 (26.7%) | |  | 539 (26.1%) | 229 (32.8%) |  |
| Not recorded | 27 (1.3%) | 20 (2.7%) |  | 30 (1.5%) | 17 (2.3%) | |  | 25 (1.2%) | 22 (3.1%) |  |
| Diabetes(Yes) | 532 (26.2%) | 229 (31.3%) | 0.030 | 501 (24.7%) | 260 (35.2%) | | <0.001 | 490 (23.7%) | 271 (38.8%) | <0.001 |
| Hypertention(Yes) | 651 (32.0%) | 266 (36.4%) | 0.001 | 634 (31.3%) | 283 (38.3%) | | <0.001 | 633 (30.7%) | 284 (40.6%) | <0.001 |
| Coronary heart disease(Yes) | 178 (8.8%) | 78 (10.7%) | 0.396 | 182 (9.0%) | 74 (10.0%) | | 0.081 | 186 (9.0%) | 70 (10.0%) | <0.001 |
| Stroke(Yes) | 117 (5.8%) | 75 (10.3%) | <0.001 | 113 (5.6%) | 79 (10.7%) | | <0.001 | 111 (5.4%) | 81 (11.6%) | <0.001 |
| Liver disease(Yes) | 120 (5.9%) | 36 (4.9%) | 0.764 | 101 (5.0%) | 55 (7.4%) | | 0.066 | 111 (5.4%) | 45 (6.4%) | 0.006 |
| laboratory data |  |  |  |  |  | |  |  |  |  |
| LogALP(IU/L) | 6.0±0.4 | 6.1±0.5 | 0.002 | 6.0±0.4 | 6.1±0.5 | | <0.001 | 6.0 ± 0.4 | 6.1±0.5 | <0.001 |
| LogAST(U/L) | 4.6±0.4 | 4.6±0.4 | 0.185 | 4.6±0.4 | 4.6±0.4 | | 0.461 | 4.6±0.4 | 4.6±0.5 | 0.896 |
| LogALT(U/L) | 4.4±0.5 | 4.3±0.6 | <0.001 | 4.4±0.5 | 4.3±0.6 | | 0.022 | 4.4±0.5 | 4.3±0.7 | <0.001 |
| AST/ALT | 1.2±0.3 | 1.3±0.4 | <0.001 | 1.2±0.3 | 1.3±0.4 | | <0.001 | 1.2±0.3 | 1.3±0.4 | <0.001 |
| LogGGT(U/L) | 4.3±0.8 | 4.4±0.9 | 0.471 | 4.3±0.8 | 4.4±0.9 | | 0.007 | 4.3±0.8 | 4.4±0.9 | 0.062 |
| ALB(g/L) | 42.2±2.8 | 41.7±3.2 | <0.001 | 42.2±2.8 | 41.6±3.3 | | <0.001 | 42.2±2.8 | 41.2±3.6 | <0.001 |
| TB(umol/L) | 11.6±4.2 | 12.3±6.3 | 0.003 | 11.8±4.6 | 11.8±5.1 | | 0.838 | 11.8±4.5 | 11.5±5.9 | 0.222 |
| TC(mmol/L) | 5.0±1.1 | 4.8±1.1 | <0.001 | 5.0±1.1 | 4.8±1.1 | | <0.001 | 5.0±1.1 | 4.8±1.2 | <0.001 |
| TG(mmol/L) | 1.7±1.1 | 1.8±1.2 | 0.288 | 1.8±1.1 | 1.7±1.2 | | 0.253 | 1.8±1.2 | 1.8±1.1 | 0.268 |
| logSCr(umol/L) | 6.3±0.4 | 6.5±0.5 | <0.001 | 6.3±0.4 | 6.5±0.6 | | <0.001 | 6.3±0.4 | 6.6±0.7 | <0.001 |
| SUA(umol/L) | 332.9±85.0 | 337.7±84.2 | 0.238 | 333.6±84.9 | 335.3±84.7 | | 0.673 | 332.6±83.9 | 341.9±90.3 | 0.049 |

Continuous variables were expressed as weighted mean ± SD and analyzed using a weighted linear regression model to determine *P* values. Categorical variables were presented as n (%) and analyzed using a weighted chi-square test to assess significance. NHANES, National Health and Nutrition Examination Survey; CERAD test: Consortium to Establish a Registry for Alzheimer's Disease test; AFT: animal fluency test; DSST: digit symbol substitution test; ALP: alkaline phosphatase; AST: aspartate aminotransferase; ALT: alanine aminotransferase; GGT: gamma-glutamyl transpeptidase; ALB: albumin; TB: total bilirubin; TC: total cholesterol; TG: triglyceride; SCr: serum creatinine; SUA: serum uric acid.
