# Supplementary material for: Non-linear association of liver enzymes with cognitive performance in the elderly: A cross-sectional study
Source: PLoS One. 2024 Jul 23;19(7):e0306839. doi: 10.1371/journal.pone.0306839 (PMC11265699; doi:10.1371/journal.pone.0306839)
Supplement: S3 Table — (DOCX) [file pone.0306839.s003.docx]

**Table S3** The associations between liver enzymes and different dimensions of cognitive performance (CERAD Test, AFT and DSST) in Model 1 and Model 2 among participants from NHANES 2011-2014 (N = 2764).

| Variable  （U/L） | CERAD Test | | AFT | | DSST | |
| --- | --- | --- | --- | --- | --- | --- |
|  | Model 1 | Model 2 | Model 1 | Model 2 | Model 1 | Model 2 |
|  | OR(95%CI) | OR(95%CI) | OR(95%CI) | OR(95%CI) | OR(95%CI) | OR(95%CI) |
| LogALP | 1.39*  (1.04-1.86) | 1.25  (0.92-1.71) | 1.90***  (1.45-2.50) | 1.62**  (1.21-2.17) | 1.73**  (1.27-2.36) | 1.21  (0.80-1.83) |
| ALP(Quartile) |  |  |  |  |  |  |
| Q1(14-54) | 1.00(Ref.) | 1.00(Ref.) | 1.00(Ref.) | 1.00(Ref.) | 1.00(Ref.) | 1.00(Ref.) |
| Q2(55-65) | 1.09  (0.77-1.55) | 1.06  (0.74-1.53) | 1.09  (0.78-1.51) | 1.06  (0.75-1.51) | 0.96  (0.67-1.37) | 0.75  (0.49-1.13) |
| Q3(66-79) | 1.28  (0.91-1.79) | 1.21  (0.84-1.73) | 1.20  (0.86-1.67) | 1.08  (0.75-1.54) | 0.96  (0.69-1.34) | 0.67*  (0.45-0.99) |
| Q4(80-336) | 1.45*  (1.04-2.03) | 1.29  (0.90-1.84) | 1.79***  (1.30-2.46) | 1.50*  (1.06-2.12) | 1.77**  (1.28-2.45) | 1.13  (0.74-1.72) |
| *P* for trend | 0.011 | 0.126 | 0.001 | 0.032 | 0.002 | 0.685 |
| LogALT | 0.68*  (0.51-0.92) | 0.70*  (0.52-0.93) | 0.80  (0.60-1.07) | 0.94  (0.71-1.25) | 0.63*  (0.43-0.92) | 0.67  (0.44-1.03) |
| ALT(Quartile) |  |  |  |  |  |  |
| Q1(5-15) | 1.00(Ref.) | 1.00(Ref.) | 1.00(Ref.) | 1.00(Ref.) | 1.00(Ref.) | 1.00(Ref.) |
| Q2(16-19) | 0.54***  (0.39-0.74) | 0.56***  (0.40-0.78) | 0.55***  (0.40-0.74) | 0.62**  (0.45-0.86) | 0.43***  (0.31-0.60) | 0.44***  (0.29-0.65) |
| Q3(20-24) | 0.43***  (0.31-0.60) | 0.46***  (0.32-0.65) | 0.50***  (0.36-0.70) | 0.62**  (0.44-0.89) | 0.31***  (0.22-0.43) | 0.31***  (0.21-0.45) |
| Q4(25-228) | 0.53***  (0.38-0.74) | 0.52***  (0.37-0.74) | 0.60**  (0.43-0.83) | 0.75  (0.53-1.07) | 0.45***  (0.32-0.62) | 0.44***  (0.29-0.67) |
| *P* for trend | <0.001 | <0.001 | 0.006 | 0.181 | <0.001 | <0.001 |
| LogAST/ALT | 2.62***  (1.86-3.67) | 2.76***  (1.93-3.96) | 1.75**  (1.24-2.48) | 1.42  (0.99-2.03) | 2.51***  (1.77-3.56) | 2.94***  (1.91-4.51) |
| AST/ALT(Quartile) |  |  |  |  |  |  |
| Q1(0.26-0.99) | 1.00(Ref.) | 1.00(Ref.) | 1.00(Ref.) | 1.00(Ref.) | 1.00(Ref.) | 1.00(Ref.) |
| Q2(1.00-1.17) | 1.10  (0.77-1.59) | 1.17  (0.79-1.73) | 1.12  (0.78-1.60) | 1.07  (0.73-1.58) | 0.87  (0.61-1.24) | 0.85  (0.56-1.30) |
| Q3(1.18-1.37) | 1.19  (0.82-1.73) | 1.27  (0.86-1.87) | 1.21  (0.84-1.73) | 1.06  (0.72-1.55) | 1.15  (0.81-1.63) | 1.21  (0.80-1.84) |
| Q4(1.38-5.12) | 2.14***  (1.51-3.04) | 2.33***  (1.61-3.38) | 1.60**  (1.13-2.26) | 1.31  (0.91-1.90) | 2.13***  (1.51-3.00) | 2.59***  (1.69-3.97) |
| *P* for trend | <0.001 | <0.001 | 0.006 | 0.152 | <0.001 | <0.001 |
| LogGGT(U/L) | 1.04  (0.91-1.19) | 0.98  (0.84-1.13) | 1.16*  (1.01-1.33) | 1.13  (0.97-1.31) | 1.13  (0.98-1.29) | 0.98  (0.82-1.18) |
| GGT(Quartile) |  |  |  |  |  |  |
| Q1(5-13) | 1.00(Ref.) | 1.00(Ref.) | 1.00(Ref.) | 1.00(Ref.) | 1.00(Ref.) | 1.00(Ref.) |
| Q2(14-18) | 1.03  (0.74-1.44) | 0.96  (0.68-1.35) | 1.18  (0.85-1.64) | 1.21  (0.86-1.69) | 0.84  (0.60-1.19) | 0.74  (0.50-1.11) |
| Q3(19-27) | 0.93  (0.66-1.31) | 0.78  (0.55-1.12) | 1.05  (0.75-1.48) | 1.00  (0.70-1.43) | 0.80  (0.57-1.11) | 0.54**  (0.36-0.80) |
| Q4(28-423) | 1.00  (0.71-1.40) | 0.81  (0.57-1.17) | 1.51*  (1.08-2.12) | 1.36  (0.95-1.93) | 1.15  (0.83-1.60) | 0.75  (0.50-1.13) |
| *P* for trend | 0.801 | 0.145 | 0.047 | 0.235 | 0.497 | 0.094 |

Weighted binary logistic regression analyses were used to caculate weighted ORs and 95% CIs. Model 1 adjusted for no covariates. Model 2 adjusted for age, gender, race, education status, and PIR. Model 3 adjusted for gender, race, age, education level, PIR, BMI, physical activity, smoking, drinking, diabetes, hypertension, stroke, coronary heart disease, liver disease, TC, TG, and SUA.CERAD Test: Consortium to Establish a Registry for Alzheimer's Disease test; AFT: animal fluency test; DSST: digit symbol substitution test. * *P* < 0.05; ** *P* < 0.01; *** *P* < 0.001.
