# Supplementary material for: Non-linear association of liver enzymes with cognitive performance in the elderly: A cross-sectional study
Source: PLoS One. 2024 Jul 23;19(7):e0306839. doi: 10.1371/journal.pone.0306839 (PMC11265699; doi:10.1371/journal.pone.0306839)
Supplement: S4 Table — (DOCX) [file pone.0306839.s004.docx]

**Table S3** Subgroup analysis of the association between quartiles of ALP and cognitive performance.

| Variable | ALP(U/L) OR(95%CI) | | | | *P* for trend | *P* for interaction |
| --- | --- | --- | --- | --- | --- | --- |
|  | Q1(14-54) | Q2(55-65) | Q3(66-79) | Q4(80-336) |  |  |
| Gender |  |  |  |  |  |  |
| Male | 1.00(Ref.) | 0.69(0.38-1.24) | 1.34(0.75-2.38) | 1.50(0.86-2.61) | 0.064 | 0.324 |
| Female | 1.00(Ref.) | 0.59(0.33-1.04) | 0.76(0.42-1.36) | 1.09(0.64-1.85) | 0.459 |  |
| Age(years) |  |  |  |  |  |  |
| ≥60 | 1.00(Ref.) | 0.50*(0.27-0.95) | 1.04(0.54-2.00) | 1.14(0.61-2.12) | 0.302 | 0.866 |
| ≥70 | 1.00(Ref.) | 0.82(0.49-1.39) | 1.05(0.64-1.75) | 1.33(0.83-2.14) | 0.165 |  |
| Race |  |  |  |  |  |  |
| Mexican American | 1.00(Ref.) | 1.97(0.53-7.33) | 1.72(0.46-6.40) | 6.11**(1.90-19.68) | 0.002 | <0.001 |
| Other Hispanic | 1.00(Ref.) | 0.66(0.21-2.06) | 1.25(0.42-3.72) | 1.54(0.55-4.33) | 0.17 |  |
| Non-Hispanic White | 1.00(Ref.) | 0.67(0.38-1.18) | 1.17(0.68-2.03) | 1.31(0.76-2.25) | 0.163 |  |
| Non-Hispanic Black | 1.00(Ref.) | 0.32**(0.17-0.63) | 0.66(0.36-1.23) | 0.69(0.40-1.20) | 0.972 |  |
| Other Race | 1.00(Ref.) | 0.83(0.26-2.72) | 0.57(0.17-1.90) | 1.14(0.32-4.04) | 0.771 |  |
| Education status |  |  |  |  |  |  |
| Below high School | 1.00(Ref.) | 1.08(0.57-2.04) | 1.24(0.66-2.33) | 2.36**(1.25-4.47) | 0.007 | 0.921 |
| High School | 1.00(Ref.) | 0.36**(0.17-0.77) | 0.80(0.37-1.74) | 0.64(0.31-1.32) | 0.559 |  |
| Above high School | 1.00(Ref.) | 0.97(0.49-1.90) | 1.31(0.68-2.52) | 1.77(0.94-3.31) | 0.059 |  |
| Physical activitity |  |  |  |  |  |  |
| No | 1.00(Ref.) | 0.51*(0.27-0.95) | 0.65(0.37-1.17) | 1.26(0.71-2.24) | 0.213 | 0.865 |
| Moderate | 1.00(Ref.) | 0.68(0.30-1.53) | 0.71(0.25-2.04) | 1.31(0.57-3.01) | 0.655 |  |
| Vigorous | 1.00(Ref.) | 0.94(0.46-1.94) | 1.97(0.98-3.94) | 1.12(0.56-2.27) | 0.229 |  |
| Alcohol |  |  |  |  |  |  |
| No | 1.00(Ref.) | 0.44*(0.22-0.91) | 0.67(0.31-1.44) | 1.24(0.61-2.53) | 0.245 | 0.351 |
| moderate | 1.00(Ref.) | 0.69(0.36-1.30) | 1.22(0.66-2.27) | 1.02(0.54-1.91) | 0.549 |  |
| heavy | 1.00(Ref.) | 0.94(0.40-2.22) | 1.17(0.51-2.72) | 1.70(0.79-3.63) | 0.157 |  |
| Smoking |  |  |  |  |  |  |
| Non-smoker | 1.00(Ref.) | 0.54*(0.31-0.94) | 0.84(0.48-1.47) | 1.11(0.63-1.97) | 0.484 | 0.641 |
| Former smoker | 1.00(Ref.) | 0.75(0.37-1.51) | 1.43(0.72-2.84) | 1.45(0.78-2.70) | 0.104 |  |
| Current smoker | 1.00(Ref.) | 1.01(0.26-3.94) | 1.06(0.32-3.48) | 1.46(0.46-4.65) | 0.443 |  |
| Hpetention |  |  |  |  |  |  |
| No | 1.00(Ref.) | 0.76(0.45-1.28) | 1.06(0.62-1.84) | 1.35(0.81-2.25) | 0.178 | 0.865 |
| Yes | 1.00(Ref.) | 0.53(0.27-1.05) | 1.06(0.56-2.02) | 1.12(0.60-2.09) | 0.293 |  |
| Diebetes |  |  |  |  |  |  |
| No | 1.00(Ref.) | 0.59*(0.36-0.97) | 1.06(0.64-1.75) | 1.14(0.70-1.86) | 0.273 | 0.835 |
| Yes | 1.00(Ref.) | 0.76(0.35-1.65) | 0.88(0.44-1.72) | 1.37(0.70-2.67) | 0.304 |  |
| Stoke |  |  |  |  |  |  |
| No | 1.00(Ref.) | 0.61*(0.40-0.95) | 0.96(0.62-1.49) | 1.10(0.73-1.65) | 0.312 | 0.169 |
| Yes | 1.00(Ref.) | 1.08(0.30-3.91) | 1.56(0.43-5.67) | 3.08(0.89-10.69) | 0.069 |  |
| Coronary heart disease |  |  |  |  |  |  |
| No | 1.00(Ref.) | 0.69(0.44-1.06) | 0.99(0.63-1.55) | 1.28(0.84-1.95) | 0.122 | 0.089 |
| Yes | 1.00(Ref.) | 0.44(0.14-1.32) | 1.62(0.44-6.04) | 0.74(0.25-2.18) | 0.916 |  |

Weighted binary logistic regression analyses were used to caculate weighted ORs and 95% CIs. Adjustment factors: gender, race, age, education level, poverty–income ratio (PIR), body mass index (BMI), physical activity, smoking, drinking, diabetes, hypertension, stroke, coronary heart disease, liver disease, TC, TG, and SUA (Model 3). * *P* < 0.05; ** *P* < 0.01.
