# Supplementary material for: Non-linear association of liver enzymes with cognitive performance in the elderly: A cross-sectional study
Source: PLoS One. 2024 Jul 23;19(7):e0306839. doi: 10.1371/journal.pone.0306839 (PMC11265699; doi:10.1371/journal.pone.0306839)
Supplement: S5 Table — (DOCX) [file pone.0306839.s005.docx]

**Table S4** Subgroup analysis of the association between quartiles of ALT and cognitive performance.

| Variable | ALT(U/L) OR(95%CI) | | | | *P* for trend | *P* for interaction |
| --- | --- | --- | --- | --- | --- | --- |
|  | Q1(5-15) | Q2(16-19) | Q3(20-24) | Q4(25-228) |  |  |
| Gender |  |  |  |  |  |  |
| Male | 1.00(Ref.) | 0.43**(0.24-0.78) | 0.39**(0.22-0.70) | 0.39**(0.22-0.69) | 0.006 | 0.542 |
| Female | 1.00(Ref.) | 0.47**(0.29-0.76) | 0.35***(0.21-0.61） | 0.79(0.46-1.34) | 0.155 |  |
| Age(years) |  |  |  |  |  |  |
| ≥60 | 1.00(Ref.) | 0.40**(0.22-0.73) | 0.36**(0.20-0.65) | 0.52*(0.29-0.96) | 0.113 | 0.219 |
| ≥70 | 1.00(Ref.) | 0.47**(0.30-0.75) | 0.41**(0.25-0.68) | 0.51*(0.31-0.86) | 0.007 |  |
| Race |  |  |  |  |  |  |
| Mexican American | 1.00(Ref.) | 1.83(0.66-5.12) | 1.68(0.54-5.27) | 0.95(0.33-2.74) | 0.871 | 0.722 |
| Other Hispanic | 1.00(Ref.) | 0.36*(0.15-0.84) | 0.24*(0.10-0.60) | 0.36*(0.15-0.86) | 0.029 |  |
| Non-Hispanic White | 1.00(Ref.) | 0.38***(0.23-0.63) | 0.27***(0.15-0.48) | 0.50*(0.29-0.85) | 0.016 |  |
| Non-Hispanic Black | 1.00(Ref.) | 0.59(0.32-1.08) | 0.94(0.54-1.64) | 1.00(0.57-1.76) | 0.787 |  |
| Other Race | 1.00(Ref.) | 0.53(0.15-1.90) | 0.95(0.29-3.12) | 0.47(0.14-1.62) | 0.42 |  |
| Education status |  |  |  |  |  |  |
| Below high School | 1.00(Ref.) | 0.61(0.32-1.16) | 0.61(0.33-1.10) | 0.51*(0.28-0.95) | 0.035 | 0.421 |
| High School | 1.00(Ref.) | 0.44*(0.23-0.87) | 0.30**(0.14-0.64) | 0.55(0.27-1.12) | 0.125 |  |
| Above high School | 1.00(Ref.) | 0.32***(0.17-0.59) | 0.29***(0.15-0.56) | 0.43**(0.23-0.80) | 0.021 |  |
| Physical activitity |  |  |  |  |  |  |
| No | 1.00(Ref.) | 0.42**(0.25-0.69) | 0.36***(0.21-0.62) | 0.47**(0.27-0.82) | 0.009 | 0.15 |
| Moderate | 1.00(Ref.) | 0.35*(0.14-0.89) | 0.39(0.15-1.03) | 0.39(0.11-1.35) | 0.175 |  |
| Vigorous | 1.00(Ref.) | 0.59(0.29-1.18) | 0.44*(0.22-0.87) | 0.70(0.36-1.34) | 0.361 |  |
| Alcohol |  |  |  |  |  |  |
| No | 1.00(Ref.) | 0.65(0.34-1.23) | 0.51(0.25-1.04) | 0.67(0.32-1.42) | 0.209 | 0.6 |
| moderate | 1.00(Ref.) | 0.33***(0.18-0.61) | 0.29***(0.16-0.52) | 0.33***(0.18-0.60) | 0.001 |  |
| heavy | 1.00(Ref.) | 0.40**(0.19-0.84) | 0.38*(0.18-0.81) | 0.65(0.29-1.46) | 0.531 |  |
| Smoking |  |  |  |  |  |  |
| Non-smoker | 1.00(Ref.) | 0.33***(0.19-0.56) | 0.36***(0.21-0.61) | 0.55*(0.31-0.96) | 0.054 | 0.815 |
| Former smoker | 1.00(Ref.) | 0.53*(0.28-0.99) | 0.39**(0.20-0.77) | 0.46*(0.24-0.91) | 0.051 |  |
| Current smoker | 1.00(Ref.) | 0.48(0.19-1.19) | 0.26*(0.08-0.79) | 0.99(0.33-2.95) | 0.427 |  |
| Hpetention |  |  |  |  |  |  |
| No | 1.00(Ref.) | 0.53**(0.32-0.85) | 0.40***(0.25-0.66) | 0.66(0.40-1.10) | 0.155 | 0.247 |
| Yes | 1.00(Ref.) | 0.35**(0.20-0.64) | 0.41**(0.22-0.76) | 0.41**(0.21-0.79) | 0.015 |  |
| Diebetes |  |  |  |  |  |  |
| No | 1.00(Ref.) | 0.47**(0.30-0.73) | 0.38***(0.24-0.61) | 0.58*(0.36-0.93) | 0.032 | 0.525 |
| Yes | 1.00(Ref.) | 0.35**(0.17-0.72) | 0.33**(0.16-0.66) | 0.36**(0.17-0.74) | 0.009 |  |
| Stoke |  |  |  |  |  |  |
| No | 1.00(Ref.) | 0.45***(0.31-0.66) | 0.36***(0.24-0.53) | 0.46***(0.30-0.70) | <0.001 | 0.014 |
| Yes | 1.00(Ref.) | 0.30(0.07-1.30) | 0.78(0.20-3.03) | 3.13(0.91-10.76) | 0.037 |  |
| Coronary heart disease |  |  |  |  |  |  |
| No | 1.00(Ref.) | 0.31*(0.10-0.94) | 0.62(0.21-1.84) | 0.30*(0.10-0.91) | 0.103 | 0.295 |
| Yes | 1.00(Ref.) | 0.49***(0.33-0.72) | 0.35***(0.23-0.54) | 0.56**(0.36-0.86) | 0.008 |  |

Weighted binary logistic regression analyses were used to caculate weighted ORs and 95% CIs. Adjustment factors: gender, race, age, education level, poverty–income ratio (PIR), body mass index (BMI), physical activity, smoking, drinking, diabetes, hypertension, stroke, coronary heart disease, liver disease, TC, TG, and SUA (Model 3). * *P* < 0.05; ** *P* < 0.01; *** *P* < 0.001.
