# Supplementary material for: Non-linear association of liver enzymes with cognitive performance in the elderly: A cross-sectional study
Source: PLoS One. 2024 Jul 23;19(7):e0306839. doi: 10.1371/journal.pone.0306839 (PMC11265699; doi:10.1371/journal.pone.0306839)
Supplement: S6 Table — (DOCX) [file pone.0306839.s006.docx]

**Table S5** Subgroup analysis of the association between quartiles of AST/ALT ratio and cognitive performance.

| Variable | AST/ALT ratio OR(95%CI) | | | | *P* for trend | *P* for interaction |
| --- | --- | --- | --- | --- | --- | --- |
|  | Q1(0.26-0.99) | Q2(1.00-1.17) | Q3(1.18-1.37) | Q4(1.38-5.12) |  |  |
| Gender |  |  |  |  |  |  |
| Male | 1.00(Ref.) | 0.98(0.54-1.78) | 1.12(0.61-2.04) | 2.42**(1.31-4.48) | 0.008 | 0.577 |
| Female | 1.00(Ref.) | 1.04(0.56-1.94) | 0.93(0.50-1.73) | 1.57(0.82-3.01) | 0.134 |  |
| Age(years) |  |  |  |  |  |  |
| ≥60 | 1.00(Ref.) | 0.93(0.53-1.66) | 0.82(0.45-1.49) | 1.85(0.96-3.57) | 0.154 | 0.069 |
| ≥70 | 1.00(Ref.) | 1.30(0.69-2.44) | 1.55(0.86-2.79) | 2.47**(1.36-4.48) | 0.001 |  |
| Race |  |  |  |  |  |  |
| Mexican American | 1.00(Ref.) | 1.02(0.39-2.65) | 2.93(1.00-8.57) | 1.46(0.50-4.25) | 0.209 | 0.974 |
| Other Hispanic | 1.00(Ref.) | 1.75(0.77-3.98) | 3.12*(1.27-7.65) | 3.13*(1.12-8.72) | 0.01 |  |
| Non-Hispanic White | 1.00(Ref.) | 0.81(0.42-1.54) | 0.78(0.42-1.47） | 2.04*(1.12-3.73) | 0.013 |  |
| Non-Hispanic Black | 1.00(Ref.) | 1.63(0.90-2.95) | 1.27(0.70-2.31) | 1.26(0.67-2.35) | 0.789 |  |
| Other Race | 1.00(Ref.) | 1.19(0.35-3.97) | 1.11(0.28-4.34) | 1.27(0.37-4.37) | 0.75 |  |
| Education status |  |  |  |  |  |  |
| Below high School | 1.00(Ref.) | 0.84(0.47-1.50) | 1.87*(1.01-3.46) | 1.24(0.64-2.44) | 0.175 | 0.415 |
| High School | 1.00(Ref.) | 1.21(0.51-2.87) | 1.01(0.42-2.45) | 3.30**(1.42-7.65) | 0.009 |  |
| Above high School | 1.00(Ref.) | 0.99(0.49-2.02) | 0.77(0.36-1.64) | 2.00*(1.01-3.95) | 0.055 |  |
| Physical activitity |  |  |  |  |  |  |
| No | 1.00(Ref.) | 1.04(0.57-1.90) | 1.16(0.62-2.15) | 1.44(0.80-2.61) | 0.17 | 0.857 |
| Moderate | 1.00(Ref.) | 0.98(0.32-3.01) | 1.61(0.64-4.03) | 5.13**(1.83-14.41) | 0.002 |  |
| Vigorous | 1.00(Ref.) | 0.38(0.57-2.18) | 0.86(0.43-1.72) | 2.45*(1.16-5.17) | 0.05 |  |
| Alcohol |  |  |  |  |  |  |
| No | 1.00(Ref.) | 0.88(0.33-2.30) | 0.86(0.34-2.16) | 2.01(0.79-5.11) | 0.06 | 0.301 |
| moderate | 1.00(Ref.) | 0.98(0.51-1.89) | 1.41(0.74-2.70) | 2.56**(1.31-5.01) | 0.003 |  |
| heavy | 1.00(Ref.) | 1.41(0.63-3.14) | 0.97(0.43-2.19) | 1.77(0.73-4.32) | 0.345 |  |
| Smoking |  |  |  |  |  |  |
| Non-smoker | 1.00(Ref.) | 0.92(0.48-1.77) | 1.09(0.57-2.07) | 2.07*(1.10-3.87) | 0.009 | 0.231 |
| Former smoker | 1.00(Ref.) | 1.29(0.65-2.57) | 1.18(0.62-2.22) | 2.69**(1.31-5.54) | 0.013 |  |
| Current smoker | 1.00(Ref.) | 0.44(0.15-1.26) | 0.38(0.12-1.20) | 0.63(0.21-1.89) | 0.56 |  |
| Hpetention |  |  |  |  |  |  |
| No | 1.00(Ref.) | 0.87(0.49-1.52) | 0.85(0.51-1.42) | 1.65(0.94-2.89) | 0.084 | 0.154 |
| Yes | 1.00(Ref.) | 1.60(0.80-3.20) | 1.58(0.76-3.27) | 2.76**(1.32-5.57) | 0.008 |  |
| Diebetes |  |  |  |  |  |  |
| No | 1.00(Ref.) | 1.00(0.57-1.78) | 1.01(0.59-1.71) | 1.80*(1.05-3.09) | 0.02 | 0.718 |
| Yes | 1.00(Ref.) | 1.14(0.61-2.14) | 1.35(0.68-2.68) | 2.93**(1.46-5.88) | 0.004 |  |
| Stoke |  |  |  |  |  |  |
| No | 1.00(Ref.) | 1.18(0.75-1.85) | 1.10(0.71-1.71) | 2.07**(1.30-3.30) | 0.003 | 0.579 |
| Yes | 1.00(Ref.) | 0.20(0.04-1.08) | 0.40(0.06-2.49) | 1.25(0.24-6.50) | 0.411 |  |
| Coronary heart disease |  |  |  |  |  |  |
| No | 1.00(Ref.) | 0.98(0.61-1.57) | 0.97(0.60-1.55) | 1.93**(1.19-3.13) | 0.006 | 0.661 |
| Yes | 1.00(Ref.) | 2.57(0.69-9.58) | 2.56(0.67-9.80) | 3.87*(1.02-14.66) | 0.1 |  |

Weighted binary logistic regression analyses were used to caculate weighted ORs and 95% CIs. Adjustment factors: gender, race, age, education level, poverty–income ratio (PIR), body mass index (BMI), physical activity, smoking, drinking, diabetes, hypertension, stroke, coronary heart disease, liver disease, TC, TG, and SUA (Model 3). * *P* < 0.05; ** *P* < 0.01.
