# Supplementary material for: Non-linear association of liver enzymes with cognitive performance in the elderly: A cross-sectional study
Source: PLoS One. 2024 Jul 23;19(7):e0306839. doi: 10.1371/journal.pone.0306839 (PMC11265699; doi:10.1371/journal.pone.0306839)
Supplement: S7 Table — (DOCX) [file pone.0306839.s007.docx]

**Table S6** Subgroup analysis of the association between quartiles of GGT and cognitive performance.

| Variable | GGT(U/L) OR(95%CI) | | | | *P* for trend | *P* for interaction |
| --- | --- | --- | --- | --- | --- | --- |
|  | Q1(5-13) | Q2(14-18) | Q3(19-27) | Q4(28-423) |  |  |
| Gender |  |  |  |  |  |  |
| Male | 1.00(Ref.) | 0.84(0.46-1.53） | 0.72(0.38-1.34) | 0.96(0.52-1.76) | 0.913 | 0.372 |
| Female | 1.00(Ref.) | 1.03(0.62-1.70) | 0.67(0.41-1.11) | 0.75(0.43-1.30) | 0.128 |  |
| Age(years) |  |  |  |  |  |  |
| ≥60 | 1.00(Ref.) | 1.00(0.53-1.87) | 0.62(0.33-1.16) | 0.75(0.41-1.39) | 0.166 | 0.752 |
| ≥70 | 1.00(Ref.) | 0.94(0.58-1.52) | 0.87(0.53-1.44) | 0.85(0.50-1.45) | 0.513 |  |
| Race |  |  |  |  |  |  |
| Mexican American | 1.00(Ref.) | 0.30*(0.10-0.94) | 0.19**(0.06-0.58) | 0.63(0.20-2.01) | 0.711 | 0.313 |
| Other Hispanic | 1.00(Ref.) | 0.54(0.22-1.32) | 0.51(0.22-1.18) | 0.30*(0.12-0.76) | 0.013 |  |
| Non-Hispanic White | 1.00(Ref.) | 1.07(0.64-1.78) | 0.81(0.46-1.41) | 0.85(0.47-1.52) | 0.373 |  |
| Non-Hispanic Black | 1.00(Ref.) | 0.93(0.49-1.78) | 0.87(0.47-1.62) | 1.01(0.54-1.90) | 0.931 |  |
| Other Race | 1.00(Ref.) | 1.22(0.32-4.72) | 1.74(0.49-6.18) | 2.09(0.43-10.07) | 0.296 |  |
| Education |  |  |  |  |  |  |
| Below high School | 1.00(Ref.) | 0.58(0.29-1.18) | 0.37**(0.19-0.71) | 0.36**(0.19-0.69) | 0.001 | 0.19 |
| High School | 1.00(Ref.) | 0.83(0.37-1.85) | 0.86(0.40-1.83) | 1.16(0.49-2.77) | 0.645 |  |
| Above high School | 1.00(Ref.) | 1.25(0.68-2.31) | 1.07(0.55-2.09) | 0.95(0.51-1.79) | 0.729 |  |
| Physical activitity |  |  |  |  |  |  |
| No | 1.00(Ref.) | 0.85(0.50-1.45) | 0.62(0.36-1.08) | 0.81(0.48-1.38) | 0.3 | 0.354 |
| Moderate | 1.00(Ref.) | 0.80(0.31-2.07) | 0.32*(0.13-0.81) | 0.62(0.24-1.63) | 0.128 |  |
| Vigorous | 1.00(Ref.) | 1.20(0.59-2.46) | 1.07(0.51-2.24) | 0.91(0.42-1.97) | 0.727 |  |
| Alcohol |  |  |  |  |  |  |
| No | 1.00(Ref.) | 1.57(0.81-3.03) | 0.75(0.35-1.63) | 1.41(0.66-3.01) | 0.97 | 0.622 |
| moderate | 1.00(Ref.) | 0.72(0.40-1.32) | 0.60(0.33-1.10) | 0.53*(0.29-0.96) | 0.031 |  |
| heavy | 1.00(Ref.) | 0.84(0.37-1.94) | 0.87(0.40-1.90) | 0.96(0.43-2.12) | 0.92 |  |
| Smoking |  |  |  |  |  |  |
| Non-smoker | 1.00(Ref.) | 1.33(0.79-2.25) | 0.89(0.50-1.56) | 0.92(0.52-1.63) | 0.421 | 0.687 |
| Former smoker | 1.00(Ref.) | 0.49*(0.25-0.96) | 0.59(0.30-1.16) | 0.67(0.35-1.27) | 0.495 |  |
| Current smoker | 1.00(Ref.) | 0.76(0.22-2.60) | 0.34(0.11-1.09) | 0.49(0.15-1.59) | 0.14 |  |
| Hpetention |  |  |  |  |  |  |
| No | 1.00(Ref.) | 0.77(0.47-1.28) | 0.77(0.46-1.28) | 0.92(0.56-1.53) | 0.821 | 0.271 |
| Yes | 1.00(Ref.) | 1.43(0.79-2.59) | 0.74(0.40-1.38) | 0.79(0.41-1.52) | 0.126 |  |
| Diebetes |  |  |  |  |  |  |
| No | 1.00(Ref.) | 1.07(0.68-1.69) | 0.79(0.49-1.26) | 0.92(0.55-1.53) | 0.433 | 0.701 |
| Yes | 1.00(Ref.) | 0.61(0.27-1.38) | 0.52(0.24-1.13) | 0.58(0.28-1.22) | 0.197 |  |
| Stoke |  |  |  |  |  |  |
| No | 1.00(Ref.) | 0.89(0.60-1.33) | 0.64*(0.42-0.97) | 0.75(0.49-1.16) | 0.083 | 0.051 |
| Yes | 1.00(Ref.) | 1.52(0.38-6.09) | 1.30(0.35-4.78) | 2.14(0.58-7.91) | 0.301 |  |
| Coronary heart disease |  |  |  |  |  |  |
| No | 1.00(Ref.) | 1.00(0.66-1.51) | 0.69(0.45-1.05) | 0.84(0.54-1.32) | 0.199 | 0.659 |
| Yes | 1.00(Ref.) | 0.79(0.23-2.68) | 1.43(0.34-6.04) | 0.79(0.21-2.93) | 0.972 |  |

Weighted binary logistic regression analyses were used to caculate weighted ORs and 95% CIs. Adjustment factors: gender, race, age, education level, poverty–income ratio (PIR), body mass index (BMI), physical activity, smoking, drinking, diabetes, hypertension, stroke, coronary heart disease, liver disease, TC, TG, and SUA (Model 3). * *P* < 0.05; ** *P* < 0.01.
