# Supplementary material for: Non-linear association of liver enzymes with cognitive performance in the elderly: A cross-sectional study
Source: PLoS One. 2024 Jul 23;19(7):e0306839. doi: 10.1371/journal.pone.0306839 (PMC11265699; doi:10.1371/journal.pone.0306839)
Supplement: S8 Table — (DOCX) [file pone.0306839.s008.docx]

**Table S7** The associations between ALP and different dimensions of cognitive performance (N = 2747, sensitivity analysis).

| Outcomes | LogALP(U/L) | ALP(U/L) OR(95%CI) | | | | *P* for trend |
| --- | --- | --- | --- | --- | --- | --- |
|  | OR(95%CI) | Q1(14-54) | Q2(55-65) | Q3(66-79) | Q4(80-166) |  |
| Global Cognitive Performance |  |  |  |  |  |  |
| Model 1 | 1.67**(1.20-2.31) | 1.00(Ref.) | 0.83(0.58-1.18) | 1.22(0.86-1.72) | 1.76**(1.25-2.46) | <0.001 |
| Model 2 | 1.28(0.88-1.86) | 1.00(Ref.) | 0.70(0.48-1.04) | 1.00(0.68-1.47) | 1.30(0.88-1.91) | 0.086 |
| Model 3 | 1.17(0.81-1.70) | 1.00(Ref.) | 0.66*(0.44-0.99) | 1.03(0.68-1.55) | 1.18(0.80-1.76) | 0.164 |
| CERAD Test |  |  |  |  |  |  |
| Model 1 | 1.32(0.97-1.79) | 1.00(Ref.) | 1.09(0.77-1.55) | 1.28(0.91-1.79) | 1.41*(1.01-1.98) | 0.018 |
| Model 2 | 1.18(0.86-1.63) | 1.00(Ref.) | 1.06(0.74-1.53) | 1.21(0.84-1.73) | 1.25(0.87-1.79) | 0.17 |
| Model 3 | 1.13(0.82-1.55) | 1.00(Ref.) | 1.05(0.73-1.51) | 1.21(0.84-1.76) | 1.19(0.83-1.70) | 0.247 |
| AFT |  |  |  |  |  |  |
| Model 1 | 1.75***(1.31-2.33) | 1.00(Ref.) | 1.09(0.78-1.51) | 1.20(0.86-1.67) | 1.71**(1.24-2.36) | 0.029 |
| Model 2 | 1.48*(1.09-2.01) | 1.00(Ref.) | 1.06(0.75-1.51) | 1.08(0.75-1.54) | 1.43*(1.01-2.03) | 0.059 |
| Model 3 | 1.38*(1.01-1.88) | 1.00(Ref.) | 1.04(0.72-1.49) | 1.12(0.77-1.61) | 1.32(0.92-1.89) | 0.129 |
| DSST |  |  |  |  |  |  |
| Model 1 | 1.59**(1.14-2.20) | 1.00(Ref.) | 0.96(0.67-1.37) | 0.96(0.69-1.34) | 1.70**(1.23-2.36) | 0.004 |
| Model 2 | 1.06(0.69-1.62) | 1.00(Ref.) | 0.75(0.49-1.13) | 0.67*(0.45-0.99) | 1.07(0.70-1.65) | 0.878 |
| Model 3 | 0.87(0.58-1.30) | 1.00(Ref.) | 0.67(0.43-1.03) | 0.64*(0.42-0.97) | 0.83(0.53-1.29) | 0.432 |

Weighted binary logistic regression analyses were used to caculate weighted ORs and 95% CIs. Model 1 adjusted for no covariates. Model 2 adjusted for age, gender, race, education status, and PIR. Model 3 adjusted for gender, race, age, education level, PIR, BMI, physical activity, smoking, drinking, diabetes, hypertension, stroke, coronary heart disease, liver disease, TC, TG, and SUA. CERAD test: Consortium to Establish a Registry for Alzheimer's Disease test; AFT: animal fluency test; DSST: digit symbol substitution test.
