# Supplementary material for: Non-linear association of liver enzymes with cognitive performance in the elderly: A cross-sectional study
Source: PLoS One. 2024 Jul 23;19(7):e0306839. doi: 10.1371/journal.pone.0306839 (PMC11265699; doi:10.1371/journal.pone.0306839)
Supplement: S9 Table — (DOCX) [file pone.0306839.s009.docx]

**Table S8** The associations between ALT and different dimensions of cognitive performance (N = 2746, sensitivity analysis).

| Outcomes | LogALT(U/L)  OR(95%CI) | ALT(U/L) OR(95%CI) | | | | *P* for trend |
| --- | --- | --- | --- | --- | --- | --- |
|  |  | Q1(5-15) | Q2(16-19) | Q3(20-24) | Q4(25-80) |  |
| Global Cognitive Performance |  |  |  |  |  |  |
| Model 1 | 0.59*(0.44-0.80) | 1.00(Ref.) | 0.42***(0.30-0.57) | 0.33***(0.24-0.45) | 0.47***(0.33-0.65) | <0.001 |
| Model 2 | 0.64*(0.47-0.88) | 1.00(Ref.) | 0.44***(0.30-0.62) | 0.36***(0.25-0.52) | 0.49***(0.33-0.73) | 0.001 |
| Model 3 | 0.66*(0.49-0.91) | 1.00(Ref.) | 0.46***(0.32-0.66) | 0.39***(0.27-0.57) | 0.51**(0.34-0.76) | 0.001 |
| CERAD Test |  |  |  |  |  |  |
| Model 1 | 0.58***(0.44-0.76) | 1.00(Ref.) | 0.54***(0.39-0.74) | 0.43***(0.31-0.60) | 0.51***(0.36-0.71) | <0.001 |
| Model 2 | 0.59***(0.45-0.76) | 1.00(Ref.) | 0.56**(0.40-0.78) | 0.46***(0.32-0.65) | 0.49***(0.35-0.70) | <0.001 |
| Model 3 | 0.59***(0.46-0.77) | 1.00(Ref.) | 0.58**(0.41-0.81) | 0.48***(0.33-0.68) | 0.50***(0.34-0.72) | <0.001 |
| AFT |  |  |  |  |  |  |
| Model 1 | 0.69**(0.52-1.89) | 1.00(Ref.) | 0.55***(0.40-0.74) | 0.50***(0.36-0.70) | 0.57**(0.41-0.80) | 0.003 |
| Model 2 | 0.81(0.62-1.05) | 1.00(Ref.) | 0.62**(0.45-0.86) | 0.62**(0.44-0.89) | 0.71(0.50-1.02) | 0.102 |
| Model 3 | 0.82(0.63-1.07) | 1.00(Ref.) | 0.65**(0.47-0.89) | 0.68*(0.47-0.98) | 0.73(0.51-1.05) | 0.146 |
| DSST |  |  |  |  |  |  |
| Model 1 | 0.47***(0.35-0.64) | 1.00(Ref.) | 0.43***(0.31-0.60) | 0.31***(0.22-0.43) | 0.41***(0.30-0.57) | <0.001 |
| Model 2 | 0.49***(0.35-0.68) | 1.00(Ref.) | 0.44***(0.29-0.65) | 0.31***(0.21-0.45) | 0.40***(0.26-0.60) | <0.001 |
| Model 3 | 0.55***(0.40-0.76) | 1.00(Ref.) | 0.48***(0.32-0.72) | 0.38***(0.26-0.56) | 0.47**(0.31-0.72) | <0.001 |

Weighted binary logistic regression analyses were used to caculate weighted ORs and 95% CIs. Model 1 adjusted for no covariates. Model 2 adjusted for age, gender, race, education status, and PIR. Model 3 adjusted for gender, race, age, education level, PIR, BMI, physical activity, smoking, drinking, diabetes, hypertension, stroke, coronary heart disease, liver disease, TC, TG, and SUA. CERAD test: Consortium to Establish a Registry for Alzheimer's Disease test; AFT: animal fluency test; DSST: digit symbol substitution test.
