# Supplementary material for: Non-linear association of liver enzymes with cognitive performance in the elderly: A cross-sectional study
Source: PLoS One. 2024 Jul 23;19(7):e0306839. doi: 10.1371/journal.pone.0306839 (PMC11265699; doi:10.1371/journal.pone.0306839)
Supplement: S10 Table — (DOCX) [file pone.0306839.s010.docx]

**Table S9** The associations between AST/ALT and different dimensions of cognitive performance (N = 2753, sensitivity analysis).

| Outcomes | LogAST/ALT  OR(95%CI) | AST/ALT OR(95%CI) | | | | *P* for trend |
| --- | --- | --- | --- | --- | --- | --- |
|  |  | Q1(0.26-0.99) | Q2(1.00-1.17) | Q3(1.18-1.37) | Q4(1.38-2.6) |  |
| Global Cognitive Performance |  |  |  |  |  |  |
| Model 1 | 2.44***(1.66-3.59) | 1.00(Ref.) | 1.06(0.72-1.55) | 1.09(0.75-1.58) | 2.03***(1.41-2.93) | <0.001 |
| Model 2 | 2.57***(1.65-4.00) | 1.00(Ref.) | 1.07(0.70-1.65) | 1.09(0.73-1.64) | 2.19***(1.45-3.31) | <0.001 |
| Model 3 | 2.40***(1.49-3.86) | 1.00(Ref.) | 1.03(0.66-1.60) | 1.05(0.69-1.62) | 1.98**(1.27-3.09) | 0.002 |
| CERAD Test |  |  |  |  |  |  |
| Model 1 | 2.56***(1.79-3.66) | 1.00(Ref.) | 1.10(0.77-1.59) | 1.19(0.82-1.73) | 2.11***(1.49-3.00) | <0.001 |
| Model 2 | 2.74***(1.87-4.01) | 1.00(Ref.) | 1.17(0.79-1.73) | 1.27(0.86-1.87) | 2.30***(1.58-3.34) | <0.001 |
| Model 3 | 2.61***(1.73-3.95) | 1.00(Ref.) | 1.16(0.78-1.73) | 1.26(0.84-1.87) | 2.19***(1.47-3.27) | <0.001 |
| AFT |  |  |  |  |  |  |
| Model 1 | 1.69**(1.18-2.43) | 1.00(Ref.) | 1.12(0.78-1.60) | 1.21(0.84-1.73) | 1.57*(1.11-2.22) | 0.009 |
| Model 2 | 1.36(0.93-1.98) | 1.00(Ref.) | 1.07(0.73-1.58) | 1.05(0.72-1.54) | 1.29(0.89-1.87) | 0.187 |
| Model 3 | 1.29(0.86-1.92) | 1.00(Ref.) | 1.08(0.72-1.61) | 1.08(0.72-1.63) | 1.18(0.79-1.76) | 0.428 |
| DSST |  |  |  |  |  |  |
| Model 1 | 2.57***(1.77-3.73) | 1.00(Ref.) | 0.87(0.61-1.24) | 1.15(0.81-1.63) | 2.12***(1.50-2.98) | <0.001 |
| Model 2 | 3.04***(1.91-4.82) | 1.00(Ref.) | 0.85(0.56-1.30) | 1.21(0.80-1.84) | 2.57***(1.67-3.95) | <0.001 |
| Model 3 | 2.60***(1.58-4.28) | 1.00(Ref.) | 0.79(0.50-1.24) | 1.16(0.74-1.81) | 2.14**(1.33-3.44) | <0.001 |

Weighted binary logistic regression analyses were used to caculate weighted ORs and 95% CIs. Model 1 adjusted for no covariates. Model 2 adjusted for age, gender, race, education status, and PIR. Model 3 adjusted for gender, race, age, education level, PIR, BMI, physical activity, smoking, drinking, diabetes, hypertension, stroke, coronary heart disease, liver disease, TC, TG, and SUA. CERAD test: Consortium to Establish a Registry for Alzheimer's Disease test; AFT: animal fluency test; DSST: digit symbol substitution test.
