# Supplementary material for: Non-linear association of liver enzymes with cognitive performance in the elderly: A cross-sectional study
Source: PLoS One. 2024 Jul 23;19(7):e0306839. doi: 10.1371/journal.pone.0306839 (PMC11265699; doi:10.1371/journal.pone.0306839)
Supplement: S11 Table — (DOCX) [file pone.0306839.s011.docx]

**Table S10** The associations between GGT and different dimensions of cognitive performance (N = 2736, sensitivity analysis).

| Outcomes | LogGGT(U/L)  OR(95%CI) | GGT(U/L) OR(95%CI) | | | | *P* for trend |
| --- | --- | --- | --- | --- | --- | --- |
|  |  | Q1(5-13) | Q2(14-18) | Q3(19-27) | Q4(28-145) |  |
| Global Cognitive Performance |  |  |  |  |  |  |
| Model 1 | 1.13(0.96-1.34) | 1.00(Ref.) | 0.99(0.70-1.38) | 0.87(0.62-1.24) | 1.16(0.82-1.63) | 0.62 |
| Model 2 | 1.03(0.85-1.25) | 1.00(Ref.) | 0.93(0.64-1.35) | 0.69(0.47-1.01) | 0.86(0.59-1.25) | 0.209 |
| Model 3 | 1.03(0.84-1.27） | 1.00(Ref.) | 0.95(0.65-1.40) | 0.72(0.48-1.07) | 0.85(0.56-1.27) | 0.218 |
| CERAD Test |  |  |  |  |  |  |
| Model 1 | 0.99(0.86-1.15) | 1.00(Ref.) | 1.03(0.74-1.44) | 0.93(0.66-1.31) | 1.11(0.79-1.55) | 0.603 |
| Model 2 | 0.92(0.78-1.08) | 1.00(Ref.) | 0.96(0.68-1.35) | 0.78(0.55-1.11) | 0.77(0.53-1.11) | 0.082 |
| Model 3 | 0.92(0.78-1.08) | 1.00(Ref.) | 0.97(0.68-1.38) | 0.78(0.55-1.12) | 0.77(0.52-1.12) | 0.083 |
| AFT |  |  |  |  |  |  |
| Model 1 | 1.17*(1.00-1.37) | 1.00(Ref.) | 1.18(0.85-1.64) | 1.05(0.75-1.48) | 1.50*(1.06-2.11) | 0.06 |
| Model 2 | 1.13(0.96-1.34) | 1.00(Ref.) | 1.20(0.85-1.68) | 1.00(0.70-1.42) | 1.34(0.94-1.91) | 0.279 |
| Model 3 | 1.13(0.95-1.36) | 1.00(Ref.) | 1.25(0.88-1.78) | 1.07(0.75-1.55) | 1.35(0.93-1.97) | 0.267 |
| DSST |  |  |  |  |  |  |
| Model 1 | 1.09(0.93-1.26) | 1.00(Ref.) | 0.84(0.60-1.19) | 0.80(0.57-1.11) | 1.11(0.79-1.55) | 0.67 |
| Model 2 | 0.92(0.76-1.12) | 1.00(Ref.) | 0.74(0.50-1.11) | 0.53**(0.36-0.80) | 0.71(0.47-1.08) | 0.054 |
| Model 3 | 0.91(0.75-1.12) | 1.00(Ref.) | 0.72(0.47-1.11) | 0.55**(0.36-0.84) | 0.67(0.43-1.06) | 0.053 |

Weighted binary logistic regression analyses were used to caculate weighted ORs and 95% CIs. Model 1 adjusted for no covariates. Model 2 adjusted for age, gender, race, education status, and PIR. Model 3 adjusted for gender, race, age, education level, PIR, BMI, physical activity, smoking, drinking, diabetes, hypertension, stroke, coronary heart disease, liver disease, TC, TG, and SUA. CERAD test: Consortium to Establish a Registry for Alzheimer's Disease test; AFT: animal fluency test; DSST: digit symbol substitution test.
