# Supplementary material for: Non-linear association of liver enzymes with cognitive performance in the elderly: A cross-sectional study
Source: PLoS One. 2024 Jul 23;19(7):e0306839. doi: 10.1371/journal.pone.0306839 (PMC11265699; doi:10.1371/journal.pone.0306839)
Supplement: Attachment — Submitted filename: Response to Reviewers.docx [file pone.0306839.s012.docx]

Dear Editor and Reviewers:

We would like to extend our most sincere appreciation to the editor and the reviewers' valuable comments on our manuscript (PONE-D-24-02163, Non-linear association of liver enzymes with cognitive performance in the elderly: A cross-sectional study). According to the valuable comments, we have improved the quality of our work, and highlighted the changes with red text in the revised manuscript. Please see below for a point-by-point response to the comments.

Reviewer #1: In this paper, the nonlinear relationship between liver enzymes and cognitive function is studied from a new Angle, the correlation and inflection point between the two are found, and the advantages and disadvantages between the two are better discussed. But there are still many problems in the article, I hope the author can modify it.

1. The BMI classification and covariates in Table 1 are inconsistent, please proofread the article and data analysis to see whether the classification is correct.
**Response:** Thanks for your constructive comment. We are very sorry for the inconsistency of BMI classification and covariates in Table 1. The classification of BMI in this paper is < 25 kg/m^2^, 25 to 30 kg/m^2^ and ≥ 30 kg/m^2^. Correspondingly, the BMI classification in Table 1 should also be < 25 kg/m^2^, 25-30 kg/m^2^ and ≥ 30 kg/m^2^. We have revised the classification of BMI in Table 1 and reanalyzed the data, as detailed in Table 1 in the revised manuscript. Thank you for your reminder.

2. Table 1 is used as the baseline table. Please make an overall description of the total number of people and whether they are sick, and modify the counting data as NO.(%) for easy reference.

**Response:** Thanks for your instructive comments. According the comments, we have make an overall description of the total number of people and whether they are sick (please see result 3.1 in the revised manuscript), and modify the counting data as NO.(%) for easy reference (please see Table 1 in the revised manuscript), thanks again!

3. It can be seen from the flow chart that the author excluded participants with missing variables, but it is still reflected in Table 1 that some participants have multiple covariables with missing values. It is suggested to delete participants with missing variables and take a complete data set for statistical analysis.

**Response:** Thank you for your valuable suggestions. We are very sorry for making it unclear. The missing variables that we exclude from the flow chart are incomplete cognitive function tests (outcome) and liver enzyme measurements (Primary Exposure), and we make sure that the major covariates are not missing.

However, only one-third of NHANES samples are serologically tested annually, limiting the total sample size. Furthermore, cognitive function data is limited to two cycles (2011-2014) of NHANES, making the available data even more precious. When excluding the missing values of other covariables, we will waste a deal of valuable information and the data of the main variables liver enzymes and cognitive performance will be incomplete and non-comprehensive.

In addition, after the deletion of the missing data of different covariables, the remaining liver enzyme and cognitive performance sample sizes differ, potentially introducing significant bias into the correlation analysis. This may lead to selective bias, affecting the reliability of the results.

In this study, we address missing values of covariables by dividing missing values of classified variables into a separate group for analysis, while converting missing values of continuous variables into classified variables. Additionally, we consult other relevant literature and refrain from excluding missing data of covariates. Such as:

1. Wang R, Wang W, Hu P, Zhang R, Dong X, Zhang D. Association of Dietary Vitamin D Intake, Serum 25(OH)D3, 25(OH)D2 with Cognitive Performance in the Elderly. Nutrients. 2021 Sep 2;13(9):3089.
2. R Cardoso B, Machado P, Steele EM. Association between ultra-processed food consumption and cognitive performance in US older adults: a cross-sectional analysis of the NHANES 2011-2014. Eur J Nutr. 2022 Dec;61(8):3975-3985.
3. Chen S, Sun X, Zhou G, Jin J, Li Z. Association between sensitivity to thyroid hormone indices and the risk of osteoarthritis: an NHANES study. Eur J Med Res. 2022 Jul 11;27(1):114.
4. Azarmanesh D, Bertone-Johnson ER, Pearlman J, Liu Z, Carbone ET. Association of the Dietary Inflammatory Index with Depressive Symptoms among Pre- and Post-Menopausal Women: Findings from the National Health and Nutrition Examination Survey (NHANES) 2005-2010. Nutrients. 2022 May 9;14(9):1980.
5. R Cardoso B, Machado P, Steele EM. Association between ultra-processed food consumption and cognitive performance in US older adults: a cross-sectional analysis of the NHANES 2011-2014. Eur J Nutr. 2022 Dec;61(8):3975-3985.

In addition, after excluding missing values of all covariables, a reanalysis of the demographic characteristics of study participants revealed that, apart from BMI, the other variables significantly associated with cognitive function remained consistent with the original data. Please refer to Tables A and B for further details.

**Table A** Characteristics of liver enzymes and the globle cognitive performance (assessed by Zscore) among participants from NHANES 2011-2014 (N = 2430).

| Variabilites | Total | Global Cognitive Performance (Zscore) | | |
| --- | --- | --- | --- | --- |
|  |  | Normal cognitive performance (N=1831) | Low cognitive performance (N=599) | *P* value |
| Gender |  |  |  | 0.2731 |
| Male | 1182 (48.6%) | 849 (46.4%) | 333 (55.6%) |  |
| Female | 1248 (51.4%) | 982 (53.6%) | 266 (44.4%) |  |
| Age(years) | 69.0 ± 6.6 | 68.7 ± 6.5 | 70.7 ± 7.0 | <0.001 |
| ≥60 | 1329 (54.7%) | 997 (54.5%) | 332 (55.4%) |  |
| ≥70 | 1101 (45.3%) | 834 (45.5%) | 267 (44.6%) |  |
| RACE |  |  |  | <0.001 |
| Mexican American | 204 (8.4%) | 130 (7.1%) | 74 (12.4%) |  |
| Other Hispanic | 240 (9.9%) | 134 (7.3%) | 106 (17.7%) |  |
| Non-Hispanic White | 1218 (50.1%) | 1031 (56.3%) | 187 (31.2%) |  |
| Non-Hispanic Black | 548 (22.6%) | 356 (19.4%) | 192 (32.1%) |  |
| Other Race | 220 (9.1%) | 180 (9.8%) | 40 (6.7%) |  |
| Education status |  |  |  | <0.001 |
| Below high school | 569 (23.4%) | 279 (15.2%) | 290 (48.4%) |  |
| High school | 578 (23.8%) | 427 (23.3%) | 151 (25.2%) |  |
| Above high school | 1283 (52.8%) | 1125 (61.4%) | 158 (26.4%) |  |
| Poverty income ratio |  |  |  | <0.001 |
| <1.3 | 706 (29.1%) | 424 (23.2%) | 282 (47.1%) |  |
| ≥1.3 | 1724 (70.9%) | 1407 (76.8%) | 317 (52.9%) |  |
| Body mass index(kg/m^2^) |  |  |  | 0.252 |
| <25 | 644 (26.5%) | 482 (26.3%) | 162 (27.0%) |  |
| 25-30 | 863 (35.5%) | 646 (35.3%) | 217 (36.2%) |  |
| ≥30 | 923 (38.0%) | 703 (38.4%) | 220 (36.7%) |  |
| Physical activity level |  |  |  | <0.001 |
| No | 985 (40.5%) | 671 (36.6%) | 314 (52.4%) |  |
| Moderate | 425 (17.5%) | 334 (18.2%) | 91 (15.2%) |  |
| Vigorous | 1020 (42.0%) | 826 (45.1%) | 194 (32.4%) |  |
| Smoking staus |  |  |  | 0.378 |
| Non-smoker | 1196 (49.2%) | 911 (49.8%) | 285 (47.6%) |  |
| Former smoker | 925 (38.1%) | 710 (38.8%) | 215 (35.9%) |  |
| Current smoker | 309 (12.7%) | 210 (11.5%) | 99 (16.5%) |  |
| Drinking status |  |  |  | <0.001 |
| No | 586 (24.1%) | 407 (22.2%) | 179 (29.9%) |  |
| Moderate | 1151 (47.4%) | 932 (50.9%) | 219 (36.6%) |  |
| Heavy | 693 (28.5%) | 492 (26.9%) | 201 (33.6%) |  |
| Diabetes(Yes) | 668 (27.5%) | 448 (24.5%) | 220 (36.7%) | <0.001 |
| Hypertention(Yes) | 800 (32.9%) | 555 (30.3%) | 245 (40.9%) | <0.001 |
| Coronary heart disease(Yes) | 224 (9.2%) | 161 (8.8%) | 63 (10.5%) | 0.295 |
| Stroke(Yes) | 163 (6.7%) | 96 (5.2%) | 67 (11.2%) | <0.001 |
| Liver disease(Yes) | 138 (5.7%) | 104 (5.7%) | 34 (5.7%) | 0.144 |
| laboratory data |  |  |  |  |
| LogALP(U/L) | 6.0±0.4 | 6.0±0.4 | 6.1±0.5 | <0.001 |
| LogAST(U/L) | 4.6±0.4 | 4.6±0.4 | 4.6±0.5 | 0.165 |
| LogALT(U/L) | 4.4±0.5 | 4.4±0.5 | 4.3±0.7 | 0.008 |
| AST/ALT | 1.2±0.3 | 1.2±0.3 | 1.3±0.4 | <0.001 |
| LogGGT(U/L) | 4.3±0.8 | 4.3±0.8 | 4.4±1.0 | 0.045 |
| ALB(g/L) | 42.1±2.9 | 42.2±2.8 | 41.4±3.4 | <0.001 |
| TB(umol/L) | 11.8±4.8 | 11.7±4.3 | 12.1±6.7 | 0.105 |
| TC (mmol/L) | 5.0±1.1 | 5.0±1.1 | 4.8±1.1 | 0.001 |
| TG(mmol/L) | 1.8±1.2 | 1.8±1.2 | 1.8±1.3 | 0.803 |
| LogSCr(umol/L) | 6.4±0.4 | 6.3±0.4 | 6.5±0.6 | <0.001 |
| SUA(umol/L) | 334.8 ± 84.9 | 335.0 ± 85.5 | 334.1 ± 81.5 | 0.854 |

Continuous variables are weighted mean ± SD and *P* values was calculated by weighted linear regression model. Categorical variables are n (%) and *P* values was calculated by weighted chi-square test. NHANES, National Health and Nutrition Examination Survey; ALP: alkaline phosphatase; AST: aspartate aminotransferase; ALT: alanine aminotransferase; GGT: gamma-glutamyl transpeptidase; ALB: albumin; TB: total bilirubin; TC: total cholesterol; TG: triglyceride; SCr: serum creatinine; SUA: serum uric acid.

**Table B** Characteristics of liver enzymes and the CERAD test, AFT, and DSST among participants from NHANES 2011-2014 (N = 2430).

| Variablites | CREAD test | | | AFT | | | DSST | | |
| --- | --- | --- | --- | --- | --- | --- | --- | --- | --- |
|  | Normal cognitive performance | Low cognitive performance | *P* value | Normal cognitive performance | Low cognitive performance | *P* value | Normal cognitive performance | Low cognitive performance | *P* value |
| Gender |  |  | <0.001 |  |  | 0.480 |  |  | 0.045 |
| Male | 773 (44.6%) | 409 (58.7%) |  | 853 (49.1%) | 329 (47.5%) |  | 821 (45.8%) | 361 (56.6%) |  |
| Female | 960 (55.4%) | 288 (41.3%) |  | 885 (50.9%) | 363 (52.5%) |  | 971 (54.2%) | 277 (43.4%) |  |
| Age(years) | 68.8 ± 6.5 | 69.8 ± 7.0 | 0.002 | 68.7 ± 6.5 | 70.2 ± 6.8 | <0.001 | 68.7 ± 6.5 | 70.9 ± 6.9 | <0.001 |
| ≥60 | 927 (53.5%) | 402 (57.7%) |  | 945 (54.4%) | 384 (55.5%) |  | 976 (54.5%) | 353 (55.3%) |  |
| ≥70 | 806 (46.5%) | 295 (42.3%) |  | 793 (45.6%) | 308 (44.5%) |  | 816 (45.5%) | 285 (44.7%) |  |
| RACE |  |  | <0.001 |  |  | <0.001 |  |  | <0.001 |
| Mexican American | 130 (7.5%) | 74 (10.6%) |  | 145 (8.3%) | 59 (8.5%) |  | 112 (6.2%) | 92 (14.4%) |  |
| Other Hispanic | 138 (8.0%) | 102 (14.6%) |  | 152 (8.7%) | 88 (12.7%) |  | 122 (6.8%) | 118 (18.5%) |  |
| Non-Hispanic White | 923 (53.3%) | 295 (42.3%) |  | 989 (56.9%) | 229 (33.1%) |  | 1048 (58.5%) | 170 (26.6%) |  |
| Non-Hispanic Black | 371 (21.4%) | 177 (25.4%) |  | 313 (18.0%) | 235 (34.0%) |  | 322 (18.0%) | 226 (35.4%) |  |
| Other Race | 171 (9.9%) | 49 (7.0%) |  | 139 (8.0%) | 81 (11.7%) |  | 188 (10.5%) | 32 (5.0%) |  |
| Education status |  |  | <0.001 |  |  | <0.001 |  |  | <0.001 |
| Below high school | 307 (17.7%) | 262 (37.6%) |  | 317 (18.2%) | 252 (36.4%) |  | 229 (12.8%) | 340 (53.3%) |  |
| high school | 402 (23.2%) | 176 (25.3%) |  | 390 (22.4%) | 188 (27.2%) |  | 424 (23.7%) | 154 (24.1%) |  |
| Above high school | 1024 (59.1%) | 259 (37.2%) |  | 1031 (59.3%) | 252 (36.4%) |  | 1139 (63.6%) | 144 (22.6%) |  |
| Poverty income ratio |  |  | <0.001 |  |  | <0.001 |  |  | <0.001 |
| <1.3 | 431 (24.9%) | 275 (39.5%) |  | 430 (24.7%) | 276 (39.9%) |  | 385 (21.5%) | 321 (50.3%) |  |
| ≥1.3 | 1302 (75.1%) | 422 (60.5%) |  | 1308 (75.3%) | 416 (60.1%) |  | 1407 (78.5%) | 317 (49.7%) |  |
| Body mass index(kg/m^2^) |  |  | 0.465 |  |  | 0.718 |  |  | 0.408 |
| <25 | 458 (26.4%) | 186 (26.7%) |  | 452 (26.0%) | 192 (27.7%) |  | 467 (26.1%) | 177 (27.7%) |  |
| 25-30 | 606 (35.0%) | 257 (36.9%) |  | 631 (36.3%) | 232 (33.5%) |  | 643 (35.9%) | 220 (34.5%) |  |
| ≥30 | 669 (38.6%) | 254 (36.4%) |  | 655 (37.7%) | 268 (38.7%) |  | 682 (38.1%) | 241 (37.8%) |  |
| Physical activity level |  |  | <0.001 |  |  | <0.001 |  |  | <0.001 |
| No | 660 (38.1%) | 325 (46.6%) |  | 633 (36.4%) | 352 (50.9%) |  | 648 (36.2%) | 337 (52.8%) |  |
| Moderate | 303 (17.5%) | 122 (17.5%) |  | 308 (17.7%) | 117 (16.9%) |  | 322 (18.0%) | 103 (16.1%) |  |
| Vigorous | 770 (44.4%) | 250 (35.9%) |  | 797 (45.9%) | 223 (32.2%) |  | 822 (45.9%) | 198 (31.0%) |  |
| Smoking status |  |  | 0.463 |  |  | 0.084 |  |  | 0.002 |
| Non-smoker | 857 (49.5%) | 339 (48.6%) |  | 851 (49.0%) | 345 (49.9%) |  | 897 (50.1%) | 299 (46.9%) |  |
| Former smoker | 671 (38.7%) | 254 (36.4%) |  | 679 (39.1%) | 246 (35.5%) |  | 702 (39.2%) | 223 (35.0%) |  |
| Current smoker | 205 (11.8%) | 104 (14.9%) |  | 208 (12.0%) | 101 (14.6%) |  | 193 (10.8%) | 116 (18.2%) |  |
| Drinking status |  |  | 0.032 |  |  | 0.001 |  |  | <0.001 |
| No | 409 (23.6%) | 177 (25.4%) |  | 389 (22.4%) | 197 (28.5%) |  | 383 (21.4%) | 203 (31.8%) |  |
| Moderate | 851 (49.1%) | 300 (43.0%) |  | 853 (49.1%) | 298 (43.1%) |  | 935 (52.2%) | 216 (33.9%) |  |
| Heavy | 473 (27.3%) | 220 (31.6%) |  | 496 (28.5%) | 197 (28.5%) |  | 474 (26.5%) | 219 (34.3%) |  |
| Diabetes(Yes) | 454 (26.2%) | 214 (30.7%) | 0.029 | 425 (24.5%) | 243 (35.1%) | <0.001 | 421 (23.5%) | 247 (38.7%) | <0.001 |
| Hypertention(Yes) | 552 (31.9%) | 248 (35.6%) | 0.002 | 544 (31.3%) | 256 (37.0%) | 0.004 | 543 (30.3%) | 257 (40.3%) | <0.001 |
| Coronary heart disease(Yes) | 154 (8.9%) | 70 (10.0%) | 0.385 | 160 (9.2%) | 64 (9.2%) | 0.825 | 161 (9.0%) | 63 (9.9%) | 0.304 |
| Stroke(Yes) | 100 (5.8%) | 63 (9.0%) | 0.003 | 99 (5.7%) | 64 (9.2%) | 0.026 | 92 (5.1%) | 71 (11.1%) | <0.001 |
| Liver disease(Yes) | 101 (5.8%) | 37 (5.3%) | 0.930 | 91 (5.2%) | 47 (6.8%) | 0.107 | 98 (5.5%) | 40 (6.3%) | 0.012 |
| laboratory data |  |  |  |  |  |  |  |  |  |
| LogALP(IU/L) | 6.0±0.4 | 6.1±0.5 | 0.006 | 6.0±0.4 | 6.1±0.5 | <0.001 | 6.0 ± 0.4 | 6.1±0.5 | <0.001 |
| LogAST(U/L) | 4.6±0.4 | 4.6±0.4 | 0.185 | 4.6±0.4 | 4.6±0.4 | 0.724 | 4.6±0.4 | 4.6±0.5 | 0.856 |
| LogALT(U/L) | 4.4±0.5 | 4.3±0.6 | 0.026 | 4.4±0.5 | 4.3±0.6 | 0.107 | 4.4±0.5 | 4.3±0.7 | 0.033 |
| AST/ALT | 1.2±0.3 | 1.3±0.4 | <0.001 | 1.2±0.3 | 1.3±0.4 | <0.001 | 1.2±0.3 | 1.3±0.4 | <0.001 |
| LogGGT(U/L) | 4.3±0.8 | 4.4±0.9 | 0.078 | 4.3±0.8 | 4.4±0.9 | 0.025 | 4.3±0.8 | 4.4±1.0 | 0.013 |
| ALB(g/L) | 42.2±2.8 | 41.8±3.1 | 0.006 | 42.2±2.8 | 41.6±3.2 | <0.001 | 42.2±2.7 | 41.3±3.5 | <0.001 |
| TB(umol/L) | 11.6±4.3 | 12.2±6.2 | 0.007 | 11.8±4.7 | 11.6±5.0 | 0.523 | 11.8±4.6 | 11.5±5.9 | 0.307 |
| TC(mmol/L) | 5.0±1.1 | 4.8±1.1 | <0.001 | 5.0±1.1 | 4.8±1.1 | <0.001 | 5.0±1.1 | 4.8±1.2 | <0.001 |
| TG(mmol/L) | 1.7±1.1 | 1.8±1.3 | 0.115 | 1.8±1.1 | 1.8±1.3 | 0.569 | 1.8±1.2 | 1.7±1.2 | 0.626 |
| logSCr(umol/L) | 6.3±0.4 | 6.5±0.5 | <0.001 | 6.4±0.4 | 6.5±0.6 | <0.001 | 6.3±0.4 | 6.6±0.7 | <0.001 |
| SUA(umol/L) | 334.5±85.2 | 335.9±84.0 | 0.718 | 334.6±85.1 | 335.9±84.2 | 0.765 | 333.5±84.1 | 342.8±89.0 | 0.057 |

Continuous variables are weighted mean ± SD and *P* values was calculated by weighted linear regression model. Categorical variables are n (%) and *P* values was calculated by weighted chi-square test.

NHANES, National Health and Nutrition Examination Survey; CERAD test: Consortium to Establish a Registry for Alzheimer's Disease test; AFT: animal fluency test; DSST: digit symbol substitution test; ALP: alkaline phosphatase; AST: aspartate aminotransferase; ALT: alanine aminotransferase; GGT: gamma-glutamyl transpeptidase; ALB: albumin; TB: total bilirubin; TC: total cholesterol; TG: triglyceride; SCr: serum creatinine; SUA: serum uric acid.

Further, after excluding missing values of all covariables, a reanalysis of the correlation between cognitive performance dimensions and liver enzyme levels among geriatric participants demonstrated that the negative association between ALP and AFT, as well as the positive association between ALT and CERAD, were no longer present in the fully adjusted model. Please refer to Tables C and D for further details.

**Table C** The associations between liver enzymes and Global Cognitive Performance among participants from NHANES 2011-2014 (N = 2430).

| Variablities(U/L) | Globe Cognitive Performance | | |
| --- | --- | --- | --- |
|  | Model 1 OR(95%CI) | Model 2 OR(95%CI) | Model 3 OR(95%CI) |
| LogALP | 1.78**(1.26-2.50) | 1.39(0.95-2.04) | 1.29(0.89-1.88) |
| ALP(Quartile) |  |  |  |
| Q1(14-54) | 1.00(Ref.) | 1.00(Ref.) | 1.00(Ref.) |
| Q2(55-65) | 0.81(0.55-1.19) | 0.69(0.45-1.06) | 0.66(0.42-1.03) |
| Q3(66-79) | 1.18(0.80-1.73) | 0.97(0.64-1.49) | 1.03(0.66-1.61) |
| Q4(80-305) | 1.84**(1.28-2.65) | 1.38(0.91-2.09) | 1.29(0.85-1.96) |
| *P* for trend | <0.001 | 0.063 | 0.092 |
| LogALT | 0.74(0.51-1.07) | 0.80(0.55-1.17) | 0.84(0.58-1.21) |
| ALT(Quartile) |  |  |  |
| Q1(5-15) | 1.00(Ref.) | 1.00(Ref.) | 1.00(Ref.) |
| Q2(16-19) | 0.36***(0.26-0.52) | 0.39***(0.27-0.58) | 0.41***(0.28-0.62) |
| Q3(20-24) | 0.34***(0.24-0.48) | 0.37***(0.25-0.55) | 0.40***(0.27-0.60) |
| Q4(25-228) | 0.50***(0.35-0.72) | 0.54**(0.36-0.82) | 0.57**(0.38-0.87) |
| *P* for trend | 0.001 | 0.012 | 0.021 |
| LogAST/ALT | 2.37***(1.58-3.55) | 2.51***(1.60-3.94) | 2.34**(1.43-3.83) |
| AST/ALT(Quartile) |  |  |  |
| Q1(0.26-0.99) | 1.00(Ref.) | 1.00(Ref.) | 1.00(Ref.) |
| Q2(1.00-1.17) | 1.05(0.69-1.59) | 1.08(0.68-1.71) | 1.02(0.64-1.64) |
| Q3(1.18-1.37) | 1.01(0.67-1.51) | 1.03(0.67-1.59) | 1.00(0.63-1.59) |
| Q4(1.38-4.20) | 1.99**(1.34-2.94) | 2.17**(1.39-3.37) | 1.93**(1.20-3.12) |
| *P* for trend | 0.001 | 0.001 | 0.006 |
| LogGGT(U/L) | 1.14(0.97-1.34) | 1.05(0.87-1.27) | 1.06(0.86-1.29） |
| GGT(Quartile) |  |  |  |
| Q1(5-13) | 1.00(Ref.) | 1.00(Ref.) | 1.00(Ref.) |
| Q2(14-18) | 1.01(0.70-1.46) | 0.97(0.65-1.44) | 0.99(0.66-1.50) |
| Q3(19-27) | 0.86(0.59-1.25) | 0.68(0.45-1.04) | 0.71(0.46-1.09) |
| Q4(28-423) | 1.14(0.78-1.65) | 0.87(0.58-1.30) | 0.87(0.56-1.35) |
| *P* for trend | 0.759 | 0.237 | 0.267 |

Weighted binary logistic regression analyses were used to caculate weighted ORs and 95% CIs. Model 1 adjusted for no covariates. Model 2 adjusted for age, gender, race, education status, and PIR. Model 3 adjusted for gender, race, age, education level, PIR, BMI, physical activity, smoking, drinking, diabetes, hypertension, stroke, coronary heart disease, liver disease, TC, TG, and SUA. * *P* < 0.05; ** *P* < 0.01; *** *P* < 0.001.

**Table D** The associations between liver enzymes and different dimensions of cognitive performance (CERAD Test, AFT and DSST) among participants from NHANES 2011-2014 (N = 2430).

| Variablities  （U/L） | CERAD Test | | | AFT | | | DSST | | |
| --- | --- | --- | --- | --- | --- | --- | --- | --- | --- |
|  | Model 1 | Model 2 | Model 3 | Model 1 | Model 2 | Model 3 | Model 1 | Model 2 | Model 3 |
|  | OR(95%CI) | OR(95%CI) | OR(95%CI) | OR(95%CI) | OR(95%CI) | OR(95%CI) | OR(95%CI) | OR(95%CI) | OR(95%CI) |
| LogALP | 1.35*  (1.01-1.81) | 1.25  (0.92-1.71) | 1.22  (0.90-1.66) | 1.67**  (1.24-2.25) | 1.37  (1.00-1.87) | 1.27  (0.94-1.73) | 1.91***  (1.38-2.66) | 1.42  (0.91-2.19) | 1.19  (0.79-1.80) |
| ALP(Quartile) |  |  |  |  |  |  |  |  |  |
| Q1(14-54) | 1.00(Ref.) | 1.00(Ref.) | 1.00(Ref.) | 1.00(Ref.) | 1.00(Ref.) | 1.00(Ref.) | 1.00(Ref.) | 1.00(Ref.) | 1.00(Ref.) |
| Q2(55-65) | 1.02  (0.71-1.47) | 0.98  (0.67-1.43) | 1.00  (0.68-1.45) | 1.05  (0.73-1.50) | 1.00  (0.68-1.45) | 0.99  (0.67-1.46) | 0.99  (0.67-1.45) | 0.78  (0.50-1.21) | 0.72  (0.46-1.14) |
| Q3(66-79) | 1.28  (0.90-1.83) | 1.23  (0.84-1.79) | 1.26  (0.85-1.85) | 1.15  (0.81-1.64) | 1.01  (0.69-1.46) | 1.02  (0.70-1.50) | 1.02  (0.71-1.44) | 0.72  (0.48-1.10) | 0.73  (0.47-1.12) |
| Q4(80-305) | 1.39  (0.98-1.97) | 1.26  (0.87-1.83) | 1.24  (0.86-1.80) | 1.65**  (1.17-2.32) | 1.32  (0.92-1.90) | 1.23  (0.85-1.78) | 1.92***  (1.36-2.71) | 1.29  (0.83-2.01) | 1.06  (0.68-1.65) |
| *P* for trend | 0.034 | 0.123 | 0.143 | 0.005 | 0.166 | 0.288 | 0.001 | 0.317 | 0.768 |
| LogALT | 0.81  (0.61-1.08) | 0.77  (0.59-1.02) | 0.78  (0.58-1.04) | 0.85  (0.64-1.14) | 0.95  (0.72-1.26) | 0.95  (0.72-1.25) | 0.71  (0.49-1,04) | 0.78  (0.51-1.17) | 0.85  (0.59-1.23) |
| ALT(Quartile) |  |  |  |  |  |  |  |  |  |
| Q1(5-15) | 1.00(Ref.) | 1.00(Ref.) | 1.00(Ref.) | 1.00(Ref.) | 1.00(Ref.) | 1.00(Ref.) | 1.00(Ref.) | 1.00(Ref.) | 1.00(Ref.) |
| Q2(16-19) | 0.67*  (0.48-0.94) | 0.71  (0.50-1.00) | 0.73  (0.51-1.04) | 0.50***  (0.36-0.70) | 0.58**  (0.41-0.82) | 0.61**  (0.43-0.86) | 0.40***  (0.28-0.56) | 0.42***  (0.27-0.64) | 0.45***  (0.29-0.70) |
| Q3(20-24) | 0.58**  (0.41-0.83) | 0.58**  (0.41-0.84) | 0.60**  (0.41-0.86) | 0.59**  (0.41-0.83) | 0.70  (0.49-1.02) | 0.75  (0.51-1.10) | 0.35***  (0.25-0.49) | 0.36***  (0.24-0.53) | 0.42***  (0.28-0.64) |
| Q4(25-228) | 0.67*  (0.48-0.96) | 0.62*  (0.43-0.89) | 0.61*  (0.42-0.89) | 0.66*  (0.47-0.93) | 0.80  (0.56-1.15) | 0.81  (0.56-1.16) | 0.50***  (0.35-0.70) | 0.53**  (0.34-0.81) | 0.61*  (0.40-0.94) |
| *P* for trend | 0.037 | 0.011 | 0.010 | 0.100 | 0.541 | 0.550 | <0.001 | 0.007 | 0.044 |
| LogAST/ALT | 2.14***  (1.50-3.06) | 2.55***  (1.76-3.70) | 2.62***  (1.76-3.91) | 1.48*  (1.03-2.13) | 1.32  (0.91-1.93) | 1.32  (0.89-1.98) | 2.07***  (1.43-2.99) | 2.30***  (1.47-3.60) | 1.98**  (1.21-3.25) |
| AST/ALT(Quartile) |  |  |  |  |  |  |  |  |  |
| Q1(0.26-0.99) | 1.00(Ref.) | 1.00(Ref.) | 1.00(Ref.) | 1.00(Ref.) | 1.00(Ref.) | 1.00(Ref.) | 1.00(Ref.) | 1.00(Ref.) | 1.00(Ref.) |
| Q2(1.00-1.17) | 1.26  (0.86-1.84) | 1.39  (0.93-2.07) | 1.39  (0.93-2.07) | 1.15  (0.79-1.68) | 1.13  (0.76-1.69) | 1.15  (0.76-1.72) | 0.93  (0.64-1.35) | 0.97  (0.62-1.53) | 0.89  (0.56-1.42) |
| Q3(1.18-1.37) | 1.20  (0.81-1.76) | 1.39  (0.93-2.08) | 1.42  (0.94-2.14) | 1.06  (0.73-1.55) | 0.99  (0.67-1.47) | 1.03  (0.69-1.56) | 1.04  (0.72-1.49) | 1.10  (0.70-1.71) | 1.04  (0.66-1.66) |
| Q4(1.38-4.20) | 1.92***  (1.33-2.78) | 2.34***  (1.59-3.44) | 2.39***  (1.59-3.60) | 1.40  (0.97-2.02) | 1.26  (0.86-1.86) | 1.23  (0.81-1.85) | 1.96***  (1.37-2.81) | 2.36***  (1.51-3.70) | 1.94**  (1.19-3.16) |
| *P* for trend | 0.001 | <0.001 | <0.001 | 0.106 | 0.352 | 0.445 | <0.001 | <0.001 | 0.004 |
| LogGGT(U/L) | 1.10  (0.95-1.28) | 1.02  (0.88-1.21) | 1.03  (0.87-1.22) | 1.14  (0.99-1.32) | 1.08  (0.93-1.26) | 1.06  (0.90-1.26) | 1.18*  (1.02-1.36) | 1.06  (0.87-1.28) | 1.05  (0.87-1.27) |
| GGT(Quartile) |  |  |  |  |  |  |  |  |  |
| Q1(5-13) | 1.00(Ref.) | 1.00(Ref.) | 1.00(Ref.) | 1.00(Ref.) | 1.00(Ref.) | 1.00(Ref.) | 1.00(Ref.) | 1.00(Ref.) | 1.00(Ref.) |
| Q2(14-18) | 1.12  (0.79-1.59) | 1.03  (0.72-1.47) | 1.05  (0.73-1.51) | 1.16  (0.82-1.64) | 1.16  (0.82-1.66) | 1.19  (0.83-1.70) | 0.93  (0.65-1.34) | 0.83  (0.54-1.26) | 0.79  (0.87-1.27) |
| Q3(19-27) | 0.99  (0.69-1.42) | 0.81  (0.56-1.17) | 0.81  (0.56-1.17) | 1.13  (0.79-1.61) | 1.04  (0.71-1.52) | 1.07  (0.73-1.57) | 0.81  (0.57-1.15) | 0.54**  (0.35-0.82) | 0.54**  (0.35-0.85) |
| Q4(28-423) | 1.19  (0.83-1.72) | 0.97  (0.66-1.42) | 0.97  (0.65-1.44) | 1.36  (0.95-1.95) | 1.16  (0.80-1.68) | 1.12  (0.76-1.65) | 1.25  (0.88-1.78) | 0.86  (0.55-1.33) | 0.81  (0.51-1.29) |
| *P* for trend | 0.537 | 0.542 | 0.510 | 0.134 | 0.628 | 0.765 | 0.350 | 0.248 | 0.223 |

Weighted binary logistic regression analyses were used to caculate weighted ORs and 95% CIs. Model 1 adjusted for no covariates. Model 2 adjusted for age, gender, race, education status, and PIR. Model 3 adjusted for gender, race, age, education level, PIR, BMI, physical activity, smoking, drinking, diabetes, hypertension, stroke, coronary heart disease, liver disease, TC, TG, and SUA.CERAD Test: Consortium to Establish a Registry for Alzheimer's Disease test; AFT: animal fluency test; DSST: digit symbol substitution test. * *P* < 0.05; ** *P* < 0.01; *** *P* < 0.001.

4. Table 1 does not provide statistical description of P value, please add.

**Response:** Thanks for your good comment. We have added the statistical description of *P* value in Table 1 (Continuous variables were expressed as weighted mean ± SD and analyzed using a weighted linear regression model to determine *P* values. Categorical variables were presented as n (%) and analyzed using a weighted chi-square test to assess significance), please see Table 1 in the revised manuscript, thanks again!

5. Table 2-5 does not conform to statistical logic. The left side is the independent variable and the right side is the dependent variable. Please revise it and combine Table 2-5 into one table.

**Response:** Thanks for your excellent suggestions. According to the suggestions, we have revised and adjusted the table format to independent variable on the left side and dependent variable on the right side. Tables 2-5 have been merged into a single table, but due to its size, it has been subsequently divided into two tables (Table 2 and Table 3). Table 2 shows the associations between liver enzymes and global cognitive performance among participants and Table 3 shows the associations between liver enzymes and different dimensions of cognitive performance (CERAD Test, AFT and DSST) among participants (please see Table 2 and Table 3 in the revised manuscript). Thanks again!

6. Please supplement the statistical method of the data in the first column of Table 2-5 in the previous data analysis (whether the independent variable is a continuous variable or a categorical variable after the four categories) and which statistical method P for trend is the P value.

**Response:** Thanks for your valuable suggestions. We used weighted binary logistic regression models and caculated ORs and 95% CIs in three model in the first column of Table 2-5 in the previous data analysis. Here, we first convert the continuous independent variable into the classified variable, and then we use weighted multivariate logical regression to test the trend of the classified variable and get the *P* value, which is the *P* for trend. Therefore, we also used weighted multivariate logistic regression as the statistical method of *P* for trend. Furthermore, if the trend test（*P* for trend） is less than 0.05, it suggests that the downward / upward trend between independent variable and dependent variable is statistically different. If the trend test（*P* for trend） is greater than 0.05, it suggests that there is no statistical difference in the downward / upward trend. That is, there is a nonlinear relationship between independent variables and dependent variables. Thanks again!

7. The author selected three models for data fitting, among which model 3 excluded the influence of confounding factors, reflecting the true correlation between liver enzymes and cognition in the presence of confounding factors. However, in most of the result descriptions, the author did not describe the contents with statistical differences in Model 3, and paid more attention to model 1. Moreover, the association between grouping variables between Q1-Q4 and cognition was not described, so the author was requested to modify the result description.

**Response:** Thank you for your valuable suggestions. We apologize for our inadequate description of the results. We have rewritten this part according to the suggestions. In the revised manuscript, we conducted a reanalysis of the relationship between liver enzymes and cognitive function, emphasizing the statistical disparities observed in Model 3 and exploring the association between grouping variables across Q1-Q4 and cognition (please see the results 3.2 description of revised manuscript). Thanks again!

8. According to Table 2-5 and S3-6, there are statistical differences in some results Q2-Q4, but P for trend is not statistically significant. Is the analysis wrong? Please refer to a published article, use the correct statistical method and do a good analysis.

**Response:** Thanks for your valuable question and good suggestions. We have reanalyzed and revised the data in corresponding Tables (please see Table2-5 in revised manuscript and Table S3-6). According to Table 2-5 and S3-6, if there are statistical differences in results Q2-Q4, the results showed that the risk of cognitive impairment (dependent variable) decreased / increased with the increase of liver enzyme level (independent variable) (Q2-Q4). Furthermore, if the trend test (*P* for trend) is less than 0.05, it suggests that the downward / upward trend is statistically different. If the trend test (*P* for trend) is greater than 0.05, it suggests that there is no statistical difference in the downward / upward trend. That is, there is a nonlinear relationship between independent variables and dependent variables.

Moreover, all our weighted logical regression analysis revealed the *P* for trend. Notably, when performing an unweighted logical regression on some of the raw data, a statistical discrepancy was observed in the *P* for trend, which contrasts with the weighted regression findings. For example, the unweighted *P* for trend of the association between quartiles of ALT and cognitive performance in non-smokers and former smokers are 0.048 and 0.008, respectively, indicating a statistically significant difference. However, the weighted *P*-values for the same trends are 0.054 and 0.051 (see Table S4), respectively, indicating no statistically significant difference. Therefore, we postulate that the discrepancy in our table is attributed to the data weighting approach.

The *P* for trend mainly plays an auxiliary role in increasing the depth of the article. In particular, when the results of other major studies are not statistically significant, the trend test results may bring additional information to make up for some of the regrets of the analysis. We also refer to some published articles, they also group the continuous independent variables into classified variables, carry out logical regression analysis, and test the trend of the results (*P* for trend).The references are as follows:

1. Yin B, Wu Z, Xia Y, Xiao S, Chen L, Li Y. Non-linear association of atherogenic index of plasma with insulin resistance and type 2 diabetes: a cross-sectional study. Cardiovasc Diabetol. 2023 Jun 29;22(1):157.
2. Shi Y, Wen M. Sex-specific differences in the effect of the atherogenic index of plasma on prediabetes and diabetes in the NHANES 2011-2018 population. Cardiovasc Diabetol. 2023 Jan 30;22(1):19.
3. Saint-Maurice PF, Troiano RP, Bassett DR Jr, Graubard BI, Carlson SA, Shiroma EJ, Fulton JE, Matthews CE. Association of Daily Step Count and Step Intensity With Mortality Among US Adults. JAMA. 2020 Mar 24;323(12):1151-1160.
4. Wei C, Tian L, Jia B, Wang M, Xiong M, Hu B, Deng C, Hou Y, Hou T, Yang X, Chen Z. Association between Serum Triglycerides and Prostate Specific Antigen (PSA) among U.S. Males: National Health and Nutrition Examination Survey (NHANES), 2003-2010. Nutrients. 2022 Mar 22;14(7):1325.
5. Chen S, Sun X, Zhou G, Jin J, Li Z. Association between sensitivity to thyroid hormone indices and the risk of osteoarthritis: an NHANES study. Eur J Med Res. 2022 Jul 11;27(1):114.

9. The discussion on logisitcs results mostly focused on the statistical results of model 1, but we mainly focused on the statistical significance of model 3 after adjusting covariates. Please adjust the relevant discussion.

**Response:** Thanks for your valuable suggestion. We apologize for our inadequate discussion of statistical significance of model 3. In the revised manuscript, we have adjusted the relevant discussion and mainly focused on the statistical significance of model 3 after adjusting covariates (please see the discussion description of revised manuscript). Thanks again!

10. The discussion of the same and different results of logistics and GAM can be added, so as to highlight the advantages of GAM model.

**Response:** Thanks for your significant suggestion. We have added the discussion of the same and different results of logistics and GAM. In our study, following covariable screening, we utilized a multivariate bivariate logistic regression model to construct three distinct models, aiming to determine the presence of a linear relationship between cognitive function and liver enzymes across all participants.

Certain findings indicated a potential correlation between elevated liver enzyme levels and a higher risk of cognitive impairment when treating liver enzymes as categorical variables. Notably, statistically significant differences were exclusively observed in the Q4 group, suggesting that the presumed linear relationship between liver enzyme levels and cognitive impairment risk may not be significant and pointing towards a potential non-linear association.

Moreover, across various models, the association between varying levels of liver enzymes and cognitive impairment risk may exhibit contrasting patterns, also indicating a nonlinear relationship and an inflection point exists between liver enzyme levels and cognitive impairment risk.

Furthermore, we utilize the GAM to fit a smoothing curve, allowing us to delve deeper into the nonlinear connection between liver enzyme levels and cognitive impairment risk. Additionally, we conduct an analysis of the threshold effect to pinpoint the turning point of the smoothing curve, thereby establishing the critical liver enzyme level threshold for maintaining normal cognitive function. please see the detailed contents in the discussion of revised manuscript. Thanks again!

We deeply appreciate your all the valuable comments and suggestions, and look forward to hearing from you regarding our submission. We would be glad to respond to any further questions and comments that you may have.

Best regards.

Yours sincerely,

Junhong Guo

E-mail:neuroguo@163.com
