# Supplementary material for: Non-linear association of liver enzymes with cognitive performance in the elderly: A cross-sectional study
Source: PLoS One. 2024 Jul 23;19(7):e0306839. doi: 10.1371/journal.pone.0306839 (PMC11265699; doi:10.1371/journal.pone.0306839)
Supplement: Attachment — Submitted filename: Response to reviewers.docx [file pone.0306839.s013.docx]

Dear Editor and Reviewers:

We would like to extend our most sincere appreciation to the editor and the reviewers' valuable comments on our manuscript (PONE-D-24-02163R1, Non-linear association of liver enzymes with cognitive performance in the elderly: A cross-sectional study). We have reviewed reference list and ensured that it is complete and correct. According to the valuable comments, we have highlighted the changes with red text in the revised manuscript. Please see below for a point-by-point response to the comments.

Reviewer #1:

1. There are unrecorded descriptions in Table 1, such as BMI, smoking, etc., but the descriptions of covariables in 2.4 Covariates are not explained, please add. Are data missing from these variables included in logisitcs and GAM? How many people are included in logisitcs and GAM?

**Response:** Thanks for your instructive comments. According the comments, we have added the descriptions of gender, BMI, smoking in Table 1 and the explaination of covariables in Table 1 (please see the corresponding contents in Result 3.1).

In this study, we address missing values of covariables by dividing missing values of classified variables into a separate group for analysis, while converting missing values of continuous variables into classified variables. Therefore, data missing from these variables were included in logisitcs analysis and 2764 individuals were included in logisitcs analysis.

In addition, GAM was employed to identify the non-linear associations between various liver enzymes and the risk of cognitive impairment. Outliers were identified and excluded for each liver enzyme marker if their values deviated by more than 4 SDs from the mean value. Therefore, 2747 individuals (see Table S8) were involved in the analysis of the associations between ALP and cognitive performance. 2746 individuals (see Table S9) were involved in the analysis of the associations between ALT and cognitive performance. 2753 individuals (see Table S10) were involved in the analysis of the associations between AST/ALT and cognitive performance. 2736 individuals (see Table S11) were involved in the analysis of the associations between GGT and cognitive performance. Thanks again!

2.3.2.1 Description of the Relationship between ALP and cognitive performance. Please describe the relationship in the order listed in the table.

**Response:** Thank you for your constructive suggestion. We have described the relationship between ALP and cognitive performance in the order listed in the Table 2 and 3 (please see the corresponding contents in Result 3.2.1). Thanks again!

3. Table 3 suggests showing the results of Model 3 directly, and the results of model 12 can be attached.

**Response:** Thank you for your valuable suggestion. We have adjusted Table 3 to show the results of Model 3 directly, and put the results of Model 1 and Model 2 in the new Table S3 (please see Table 3 and Table S3 in the revised manuscript).

4. As far as I know, different dimensions of cognitive performance include CERAD-IR, Consortium to Establish a Registry for Alzheimer's Disease Immediate Recall, CERAD-DR, Consortium to Establish a Registry for Alzheimer's Disease Delayed Recall, AFT, animal fluency test; DSST, digit symbol substitution test.Why are there only three dimensions in this article?

**Response:** Thank you for your valuable questions. We are very sorry for making it unclear in the text. In our study, the cognitive function of participants aged ≥ 60 was assessed using three tests including word learning and recall modules from the consortium to establish a registry for Alzheimer's disease (CERAD) test, the animal fluency test (AFT), and the digit symbol substitution test (DSST).

CERAD test evaluates the capacity to acquire and retain new verbal information, comprising three consecutive learning trials (CERAD-IR, consortium to establish a registry for Alzheimer's Disease immediate recall) and a delayed recall task (CERAD-DR, consortium to establish a registry for Alzheimer's Disease delayed recall). During the learning trials (CERAD-IR), participants were prompted to recall as many words as possible immediately after being instructed to articulate 10 unrelated words. The delayed word-recall task (CERAD-DR) occurred after the completion of the AFT and DSST. The overall score of CERAD test was determined by summing the results of the three immediate tests (CERAD-IR) and delayed recall test (CERAD-DR). And we have revised the corresponding contents in the revised manuscript. Thanks again!

We deeply appreciate your all the valuable comments and suggestions, and look forward to hearing from you regarding our submission. We would be glad to respond to any further questions and comments that you may have.

Best regards.

Yours sincerely,

Junhong Guo

E-mail:neuroguo@163.com
